# Exploration of the Canyon-Incised Continental Margin of the Northeastern United States Reveals Dynamic Habitats and Diverse Communities

**DOI:** 10.1371/journal.pone.0139904

**Published:** 2015-10-28

**Authors:** Andrea M. Quattrini, Martha S. Nizinski, Jason D. Chaytor, Amanda W. J. Demopoulos, E. Brendan Roark, Scott C. France, Jon A. Moore, Taylor Heyl, Peter J. Auster, Brian Kinlan, Carolyn Ruppel, Kelley P. Elliott, Brian R.C. Kennedy, Elizabeth Lobecker, Adam Skarke, Timothy M. Shank

**Affiliations:** 1 Cherokee Nations Technology Solutions, Contracted to the Wetland and Aquatic Research Center, US Geological Survey, Gainesville, FL, United States of America; 2 NOAA, NMFS, National Systematics Laboratory, National Museum of Natural History, Washington, DC, United States of America; 3 USGS Woods Hole Coastal and Marine Science Center, Woods Hole, MA, United States of America; 4 Wetland and Aquatic Research Center, US Geological Survey, Gainesville, FL, United States of America; 5 Department of Geography, Texas A&M University, College Station, TX, United States of America; 6 Department of Biology, University of Louisiana at Lafayette, Lafayette, LA, United States of America; 7 Wilkes Honors College, Florida Atlantic University, Jupiter, FL, United States of America; 8 Harbor Branch Oceanographic Institute, FAU, Ft. Pierce, FL, United States of America; 9 Biology Department, Woods Hole Oceanographic Institution, Woods Hole, MA, United States of America; 10 Department of Marine Sciences and Northeast Undersea Research Technology and Education Center, University of Connecticut, Groton, CT, United States of America; 11 Sea Research Foundation, Mystic Aquarium, Mystic, CT, United States of America; 12 NOAA National Ocean Service, Silver Spring, MD, United States of America; 13 NOAA Office of Ocean Exploration and Research, Silver Spring, MD, United States of America; 14 NOAA Office of Ocean Exploration and Research, Narragansett, RI, United States of America; 15 Earth Resources Technology Corporation, Contracted to the NOAA Office of Ocean Exploration and Research, Durham, NH, United States of America; 16 Department of Geosciences, Mississippi State University, Mississippi State, MS, United States of America; Università di Genova, ITALY

## Abstract

The continental margin off the northeastern United States (NEUS) contains numerous, topographically complex features that increase habitat heterogeneity across the region. However, the majority of these rugged features have never been surveyed, particularly using direct observations. During summer 2013, 31 Remotely-Operated Vehicle (ROV) dives were conducted from 494 to 3271 m depth across a variety of seafloor features to document communities and to infer geological processes that produced such features. The ROV surveyed six broad-scale habitat features, consisting of shelf-breaching canyons, slope-sourced canyons, inter-canyon areas, open-slope/landslide-scar areas, hydrocarbon seeps, and Mytilus Seamount. Four previously unknown chemosynthetic communities dominated by *Bathymodiolus* mussels were documented. Seafloor methane hydrate was observed at two seep sites. Multivariate analyses indicated that depth and broad-scale habitat significantly influenced megafaunal coral (58 taxa), demersal fish (69 taxa), and decapod crustacean (34 taxa) assemblages. Species richness of fishes and crustaceans significantly declined with depth, while there was no relationship between coral richness and depth. Turnover in assemblage structure occurred on the middle to lower slope at the approximate boundaries of water masses found previously in the region. Coral species richness was also an important variable explaining variation in fish and crustacean assemblages. Coral diversity may serve as an indicator of habitat suitability and variation in available niche diversity for these taxonomic groups. Our surveys added 24 putative coral species and three fishes to the known regional fauna, including the black coral *Telopathes magna*, the octocoral *Metallogorgia melanotrichos* and the fishes *Gaidropsarus argentatus*, *Guttigadus latifrons*, and *Lepidion guentheri*. Marine litter was observed on 81% of the dives, with at least 12 coral colonies entangled in debris. While initial exploration revealed the NEUS region to be both geologically dynamic and biologically diverse, further research into the abiotic conditions and the biotic interactions that influence species abundance and distribution is needed.

## Introduction

The continental margin of the northeastern United States (NEUS) region (including the Gulf of Maine, Georges Bank to Cape Hatteras, NC) contains numerous, topographically complex features, including submarine canyons, landslide-scars, seamounts, and hydrocarbon (cold) seeps. These features increase habitat heterogeneity in the deep sea, thus influencing the distribution and abundance of deep-sea fauna and increasing both local and regional scale diversity [1–3]. Yet, it is unclear whether all complex features (and non-complex features) are functionally equivalent to one another for various taxonomic groups. The diversity of seafloor features that exist in the NEUS region across a broad depth range provide an exemplary setting to further our understanding of how habitat features and other environmental conditions influence benthic communities in the deep sea. Additionally, documenting patterns of biodiversity along the NEUS continental margin region is critical as the fisheries, energy, and minerals sectors seek to expand into deeper depths [4]. Thus, seafloor communities along the NEUS continental slope could be highly impacted by current and future anthropogenic disturbances.

The geology of the NEUS margin and the broad range of chemical, physical and biological processes acting upon it control this rugged, complexity of the deep sea floor. As the type-example of passive continental margins [5], the regional geologic history and geomorphology of the NEUS margin has been intensely studied. However, the processes occurring at the local scale of individual geomorphic features (e.g., canyons, channels, interfluves, and landslide scars) are less understood. For example the roles of the underlying lithologies, modern erosional and depositional processes, and forcing mechanisms in shaping the margin remain largely unconstrained.

Some of the most pervasive, complex features along the NEUS continental margin include submarine canyons. Approximately 40 shelf-breaching and numerous slope-sourced canyons exist on the NEUS continental margin [6]. Submarine canyons are topographically and oceanographically complex features, linking the upper continental shelf to the abyssal plain. These features serve as major conduits for the transport of sediments [7–9] and particulate organic matter [8, 10, 11]. The concentration of organic matter and nutrient-rich sediments in submarine canyons combined with strong currents and turbidity flows [4, 12–14] can support and concentrate suspension and deposit feeding organisms, commercially and ecologically important fishes, and dense invertebrate assemblages. Specifically, oceanographic conditions, food quality and food availability in canyons provide favorable areas for recruitment of mega- and macro-faunal species [15–20]. A variety of hard-substrate features, such as steep walls, rocky outcrops, and debris fields, further contribute to the habitat complexity within submarine canyons across varying depths [21–23]. The presence of such a diverse and variable array of environmental factors has led to the hypothesis that submarine canyons support higher biodiversity and biomass compared with areas on the adjacent continental slope [11, 15, 21, 24, 25]. Additionally, submarine canyons appear to be essential habitat for some species [26], including fishes [17, 27]. In fact, the rugged topography of canyons provides refuge to commercially important fishes from bottom fishing activity [27]. However, it is not uncommon for fisheries to operate within canyon environments using fixed gears such as traps and longlines [28].

Despite the observation that canyon ecosystems are among the most productive and diverse areas in deep waters worldwide [29], few have been mapped extensively using high- resolution multibeam sonar or investigated using fine-scale direct observations. As evidenced along the NEUS continental margin, only a small portion (~1%) of the canyons (Hudson, Baltimore, Oceanographer, Lydonia) has been studied more thoroughly [30–34]. These early studies documented general patterns of habitat variability and dominant fauna, noting the presence of cold-water corals, and laid the groundwork for future research.

Cold-water (or deep-sea) corals, including scleractinians (stony corals), stylasterid hydrocorals (lace corals), antipatharians (black corals), and octocorals (soft corals, sea fans, sea pens), have been documented previously in submarine canyons, on seamounts, and in other deep-sea habitats. These foundation species either create massive reef frameworks (such as *Lophelia pertusa*) or individually colonize existing soft or hard substrates, further contributing to the overall habitat heterogeneity in the deep sea. A diverse assemblage of invertebrates and fishes [35–42], some of which are obligate associates [41, 42], are often found with these sessile coral species. Cold-water corals are known to serve as food resources for certain echinoderms [43] and nursery habitats for some fishes [44]. In submarine canyons, cold-water corals have been observed on canyon walls in dense aggregations [19, 45], which may be driven, in part, by the quality and availability of food [19].

Another important component enhancing environmental heterogeneity in deep-sea environments is hydrocarbon seepage [2]. Recent investigations on the NEUS continental margin documented approximately 570 gas plumes escaping from the seafloor, yet <1% of these have been ground truthed using direct observations [46]. In the region, hydrocarbon (cold) seeps occur on promontories overlooking canyon heads, ridges within canyons, and on the open, upper to middle slope [46]. Here, *Bathymodiolus* mussels have been documented [46], serving as foundation species by creating habitat and modifying both the physical and chemical environment [47]. Thus, *Bathymodiolus* mussels can promote the colonization of other fauna that are often endemic to or dependent upon chemosynthetic habitats [47–49]. Additionally, authigenic carbonates that form through microbial processes facilitate the colonization of other foundation species, including cold-water corals and sponges [48].

Data on local-scale geologic processes, the benthic communities, and the relationships of these benthic communities to substrate type and geologic processes along the rugged NEUS continental margin are limited. Few direct observations utilizing remotely-operated or human-occupied vehicles exist in the region (but see [7, 28, 33, 34, 50]); yet, submersibles provide the most in-depth views of fauna inhabiting features that are difficult to sample or image with other types of equipment. In the summer of 2013, an expedition off the NEUS using a remotely-operated vehicle provided the opportunity to gain insight into the geology, oceanography, habitat diversity, and biodiversity of the NEUS continental margin across a broad depth range, with a particular focus on submarine canyons. The telepresence-enabled expedition (http://oceanexplorer.noaa.gov/okeanos/welcome.html) explored diverse habitats while engaging more than 40 scientists onshore across national and international boundaries.

In the present study, we 1) describe the environmental parameters measured within a variety of seafloor features, including submarine canyons, inter-canyon areas, open-slope/landslide-scar areas, cold seeps, and a seamount; 2) assess the biodiversity and community structure of corals, demersal fishes and megafaunal decapod crustaceans; and 3) document the occurrence of marine litter throughout the region. Specifically, we utilized information gathered from image data to test the hypotheses that benthic assemblage structure and species richness differ with environmental conditions, particularly broad-scale habitat (seafloor) features and depth. With this dataset, we were able to compare patterns in species richness and assemblage structure among these three taxonomic groups.

## Materials and Methods

### Ethics Statement

The NEUS Canyons Expedition was conducted within the exclusive economic (EEZ) zone of the United States. No permits were necessary as no geologic or biological specimens were collected and research was not conducted within the boundaries of a national marine sanctuary, marine national monument or other protected area. This expedition did not involve the study of marine mammals or endangered species, did not involve taking commercial quantities of marine resources, and did not involve ocean dumping research.

In order to conduct marine scientific research within the U.S. EEZ, work funded, authorized and/or conducted by NOAA must be compliant with the National Environmental Policy Act (NEPA). In compliance with NOAA Administrative Order (NAO) 216–6 and NEPA, this project was determined to have no potential to result in any lasting changes to the environment. This project was determined to be of limited size and magnitude with only short-term effects on the environment; any cumulative effects were negligible. As such, the requirements of NAO 216–6 and NEPA were met; the marine scientific research for the project was authorized.

### ROV Surveys

**Fig 1:**
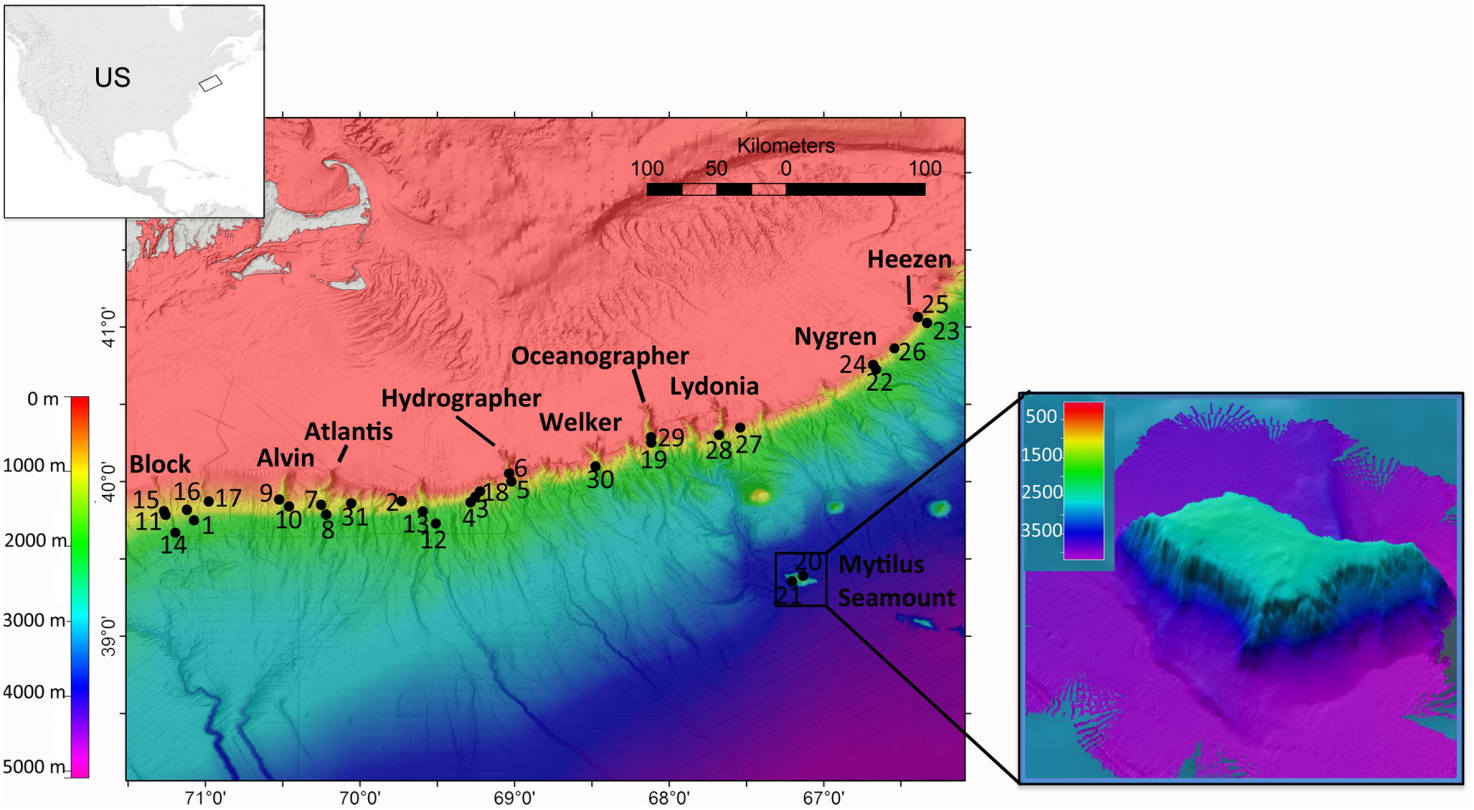
Dive locations (numbered) for the 2013 NEUS. Canyons Expedition. All canyons and seamounts surveyed during the expedition are labeled. Mytilus Seamount bathymetry is at 50 m resolution.

Exploration along the NEUS continental margin (Fig 1) was conducted during a cruise of the NOAA ship *Okeanos Explorer* (EX 1304, 9 July–16 August 2013). The 6,000 m-rated dual-body system, including the remotely-operated vehicle (ROV) *Deep Discoverer* (*D2*) and *Seirios* camera sled, was deployed during a series of daytime dives. *Seirios* was tethered to the ship with a standard oceanographic armored, fiber-optic cable (1.73 cm diameter). The ROV *D2* was linked to *Seirios* with a neutrally buoyant tether, isolating the ROV from surface ship’s motion and allowing precise maneuvering in precipitous topography.

The ROV *D2* carried two maneuverable and four fixed video cameras with two high-definition video cameras used principally for scientific observations. Paired lasers (10 cm apart) were mounted on the fixed, high-definition video camera. Lighting consisted of 16 LED lamps (96,000 lumens total) with eight of these on four hydraulically positioned booms. *Seirios* carried five video cameras (two in high-definition and maneuverable) and six HMI lamps (72,000 lumens total). High-definition video was transmitted from both vehicles in HD-SDI 1080i format. The *D2* has an integrated navigation and control system that allows precision auto-pilot flight to maintain heading and altitude along straight transits and maintain position over features of interest. Navigational sensors, including a fiber-optic gyroscope and Doppler velocity log, produced fine-scale data on direction and transit distances over the seafloor. High-speed fiber-optic communications over the vehicle tether carried video, vehicle control, and sensor telemetry. Sea-bird 911+ conductivity-temperature-depth (CTD) loggers with dissolved oxygen (DO) sensors were also attached to both *D2* and *Seirios*.

ROV surveys were exploratory to target different seafloor features over a broad depth range. Six broad-scale habitat features were surveyed, including shelf-breaching canyons, slope-sourced canyons, cold seeps, inter-canyon areas, open slope/landslide scars, and a seamount, specifically Mytilus Seamount. General dive locations were chosen based on more than 90,000 km^2^ of seafloor mapping data collected by four NOAA ships over three field seasons. Within shelf-breaching and slope canyons, directional tracks were selected based on elevated acoustic impedance and high-slope angles (>30°) observed in bathymetry and backscatter data collected with multibeam sonar (Kongsberg EM 302, 30 kHz, 0.5 X 1 degree sonar) on the *Okeanos Explorer*. Cold seep dive locations were chosen based on locations where multibeam backscatter data documented the emission of bubble streams rising from the seafloor [46]. Additionally, the ROV *D2* surveyed two sites in inter-canyon areas and five sites on the open slope that were characterized as potential geohazards (e.g., submarine landslide areas). Mytilus Seamount also was targeted because it is the deepest (~2500 m at the summit) and least explored seamount in the New England Seamount Chain in the U.S. EEZ.

The ROV *D2* traversed the seafloor at a speed of approximately ~ 0.1–0.3 knots (1 knot = 0.514 m s^-1^). *Seirios* surveyed approximately 10 m above and behind *D2*, providing background lighting to visualize features as well as situational awareness for the pilots and scientists. The *Okeanos Explorer* followed the vehicles using dynamic positioning and tracked vehicle position relative to the ship with an ultra-short baseline tracking system. When surveying canyon walls, *D2* was typically deployed at the base of a wall on one side of the canyon, and then traversed up and along the wall as close to the bottom as practical. As the ROV traversed the seafloor, the cameras were generally set on wide-angle view to document habitat features, species occurrences, and behaviors. Zooms were frequently conducted to obtain detailed imagery of each morphotype to facilitate taxonomic identification.

### Telepresence Capabilities

*Okeanos Explorer* is equipped with high-speed communication capabilities to enable scientists on the shore to participate in ship operations in real time via telepresence. Scientists (and anyone with an Internet connection) were able to observe live video feeds (yielding 660,000 visits) from the ROV *D2* (http://oceanexplorer.noaa.gov/okeanos/media/exstream/exstream.html). Scientists were also able to participate during the dives in real-time via a private Internet chat room and satellite teleconference line (see http://oceanexplorer.noaa.gov/okeanos/collaboration-tools/welcome.html). Eight to 35 scientists on shore engaged in the daily ROV dives, providing taxonomic expertise and scientific discussion.

### Data Analyses

CTD data collected throughout each dive were averaged to obtain mean temperature, salinity, and DO (ml L^-1^) while at depth. However, during Leg 1 (dives 1–16) of the expedition, the CTD and DO sensors were not functioning properly. Therefore, temperature, salinity, and DO concentrations were obtained for 10 of the dives with shipboard CTD (SBE 911+) deployments at each site.

Corals, demersal fishes, and decapod crustaceans were identified to the lowest possible taxon or morphotype using taxonomic keys and through collaborative exchange with taxonomic experts. Taxonomic authorities for each taxon are presented in supplementary tables. We focused on these three taxonomic groups for the present study because these taxa were conspicuous, amenable to identification via imaging (e.g., [37, 40, 42]), and some are relevant to management concern in the region [51]. The presence or absence of morphospecies within these taxonomic groups was documented during each dive via analysis of frame grabs and video clips from high-definition video. Because no voucher specimens were collected during this expedition, photographic identifications were conservative. We recognize that specimen collections are critical to ground truth identifications from imagery. Often, identifications were made to either the level of genus or morphotype. Diagnostic characters were not always apparent, particularly for invertebrates and some fishes (e.g., ophidiiform, gadiiformes).

Species richness (the raw number of different species or morphotypes observed) was estimated for each focal taxonomic group. Species richness was determined for each dive (alpha diversity) and each canyon. Species accumulation curves were generated to estimate how well each of the focal taxa were documented within the region (gamma diversity; EstimateS v 7.5, [52]). The resampling-based SOBS (observed number of species, *Mao Tau*) method was used to generate expected number of species and 95% confidence intervals for each dive [53–54]. Linear regressions of species richness (log X+1 transformed) with depth (log X+1 transformed) and dive distance (log X+1 transformed) were also calculated (SigmaStat v 3.0) for each taxonomic group.

Multivariate analyses were performed (Primer 6, [55–56]) to determine whether benthic assemblages differed with varying environmental conditions (see S1 Table). The Sorensen’s Index of Similarity (beta diversity), based on presence/absence data, was calculated between all pairs of dives separately for fishes, corals, and crustaceans. A non-metric multidimensional scaling (MDS) ordination plot and a dendrogram based on hierarchical clustering of group average linking were created from each similarity matrix. Significantly dissimilar clusters defined by a SIMPROF permutation test were overlain onto the MDS plots to determine whether any significant clusters corresponded to specific depth zones. The SIMPER routine was used to determine which taxa were contributing to the significant dissimilarity in assemblage structure in clusters defined by SIMPROF. The top ranking species that contributed to the majority of the average dissimilarity in assemblage structure are reported here. Distance-based Linear Modeling (DistLM) was performed using PERMANOVA+ (Primer 6 add-on package; [57]) to examine what environmental factors influenced taxonomic assemblages. The following factors were included: depth (log X+1 transformed), salinity (log transformed), DO (log transformed), over- the-ground distance covered by the *D2* (log X+1 transformed), broad-scale habitat feature (coded as nominal, binary categories, and then grouped as the indicator ‘habitat’), and coral species richness (for fish and crustacean analyses) (see S1 Table). We did not include temperature in this analysis because it was highly correlated with depth (Pearson Correlation, *r* = -0.96). Although salinity (*r =* 0.90) was also correlated with depth, this variable was retained in analyses (as per [57]). Seven of 31 dives were not included in DistLM analyses because CTD data were not available. Marginal tests (999 permutations) were performed to determine the explanatory power of each environmental variable on assemblage structure. The BEST selection procedure, combined with the Akaike Information Criterion (AIC, [58]), was used to ascertain the optimal combination of environmental variables that explained the majority of the variation in assemblage structure.

Abundances of dominant and characteristic species for each of the three focal groups were calculated. The fishes *Synaphobranchus* spp. and *Neocyttus helgae*, the corals *Paragorgia* spp. and *Primnoa*? *resedaeformis*, and the crustacean *Chaceon quinquedens* were enumerated during dives on which they were observed. These species were chosen for abundance analyses because they could be most accurately enumerated, can be compared to data from other regions (e.g., [59]) and/or represent species of interest for conservation and management purposes [51]. Abundances were estimated per dive as the number of individuals observed divided by the product of the total over-the-ground distance covered by the ROV and an estimated, average field of view (4.3 m).

Other observational data were noted from the video. The generalized lithology and age of geological features were inferred based on previous work [60–65] in the region using observed texture, physical properties and depth-linked stratigraphic position. Qualitative assessments of the sediment composition, the magnitude and contributing processes of substrate erosion, and sediment deposition were also made. Marine litter was enumerated during each dive and quantities were estimated as above.

## Results and Discussion

Thirty-one ROV dives, resulting in 201.6 hours of bottom time, were conducted at depths ranging from 494 to 3271 m. The over-the-ground distance covered by the ROV varied across dives (300–2200 m), but the observation time on bottom was approximately the same (5–7 hours per dive; Table 1, S1 Table). Observational effort did not bias this dataset as there were no significant relationships between ROV distance and alpha diversity (linear regression, p>0.05) and beta diversity (DISTLM, marginal tests, p = 0.018 to 0.385, α = 0.01, Bonferroni Correction) for any taxonomic group (Table 2, S1 Fig) (following [66–67]).

**Table 1:** Total number of morphospecies observed across six broad-scale habitat features for three taxonomic groups. Depth range and total distance travelled per area are included. Dive numbers are included.

Site	No. Dives	Dive Numbers	Depth Range (m)	Total Distance^a^ (m)	Coral	Fish	Crustacean
**Shelf-breaching Canyon**							
Hydrographer	2	5, 6	580–1423	1460	20	18	9
Atlantis	2	7, 8	885–1794	1400	24	16	10
Alvin	2	9, 10	846–1110	1330	16	26	10
Block	3	11, 14, 15	1044–2135	1575	24	20	14
Oceanographer	2	19, 29	983–1248	1430	24	14	11
Nygren	2	22, 24	678–1590	1435	28	24	11
Heezen	2	23, 25	703–1723	1015	18	24	10
Lydonia	1	28	1135–1239	300	17	12	7
**Slope Canyon**							
Un-Named	1	16	1018–1121	500	10	14	5
Un-Named	1	18	1025–1139	560	18	13	10
Welker	1	30	1377–1445	350	21	7	5
**Seamount**							
Mytilus	2	20, 21	2592–3271	1620	18	8	3
**Inter-canyon Slope**							
Nygren-Heezen	1	26	497–824	1200	12	19	10
Lydonia-Powell	1	27	494–663	1180	0	13	6
**Cold Seep**							
Veatch Seeps	1	13	1409–1423	350	5	6	2
NE Seep 2	1	3	1053–1139	975	0	10	1
NE Seep 3	1	4	1410–1484	1075	0	11	2
**Open Slope/Landslide Scar**							
USGS1	1	17	647–784	2200	0	10	4
USGS2/Veatch	1	12	1967–2026	1050	6	9	2
USGS3	1	1	1610–1880	965	13	9	2
USGS4	1	2	555–609	2100	0	11	6
USGS5	1	31	778–899	2140	0	15	7

^a^ Straight-line distance travelled over ground was approximated using the measuring tool in ArcMap.

**Table 2:** DistLM results. Marginal test results followed by results from the BEST model using the AIC criterion. P-values in bold are significant (Bonferroni Adjustment, α = 0.01).

	Corals			Fishes			Crustaceans		
Environ. Factor	Pseudo-F	Prop. Var.	p	Pseudo-F	Prop. Var.	p	Pseudo-F	Prop. Var.	p
Depth (m)	9.89	0.35	**0.001**	7.01	0.24	**0.001**	8.54	0.28	**0.001**
Salinity	8.48	0.32	**0.001**	5.81	0.21	**0.001**	7.74	0.26	**0.001**
Distance (m)	1.02	0.05	0.385	1.21	0.05	0.282	2.89	0.11	0.013
DO (ml/L)	3.45	0.16	0.019	2.15	0.09	0.048	1.25	0.05	0.270
Habitat	4.35	0.45	**0.005**	2.46	0.41	**0.007**	2.41	0.40	**0.001**
Coral Richness	—	—	—	2.28	0.09	0.022	3.45	0.13	**0.004**
AIC		135.85			178.44			178.49	
R^2^		0.71			0.71			0.67	
Model	All except Distance			All Variables			All except Distance and DO		

**Fig 2:**
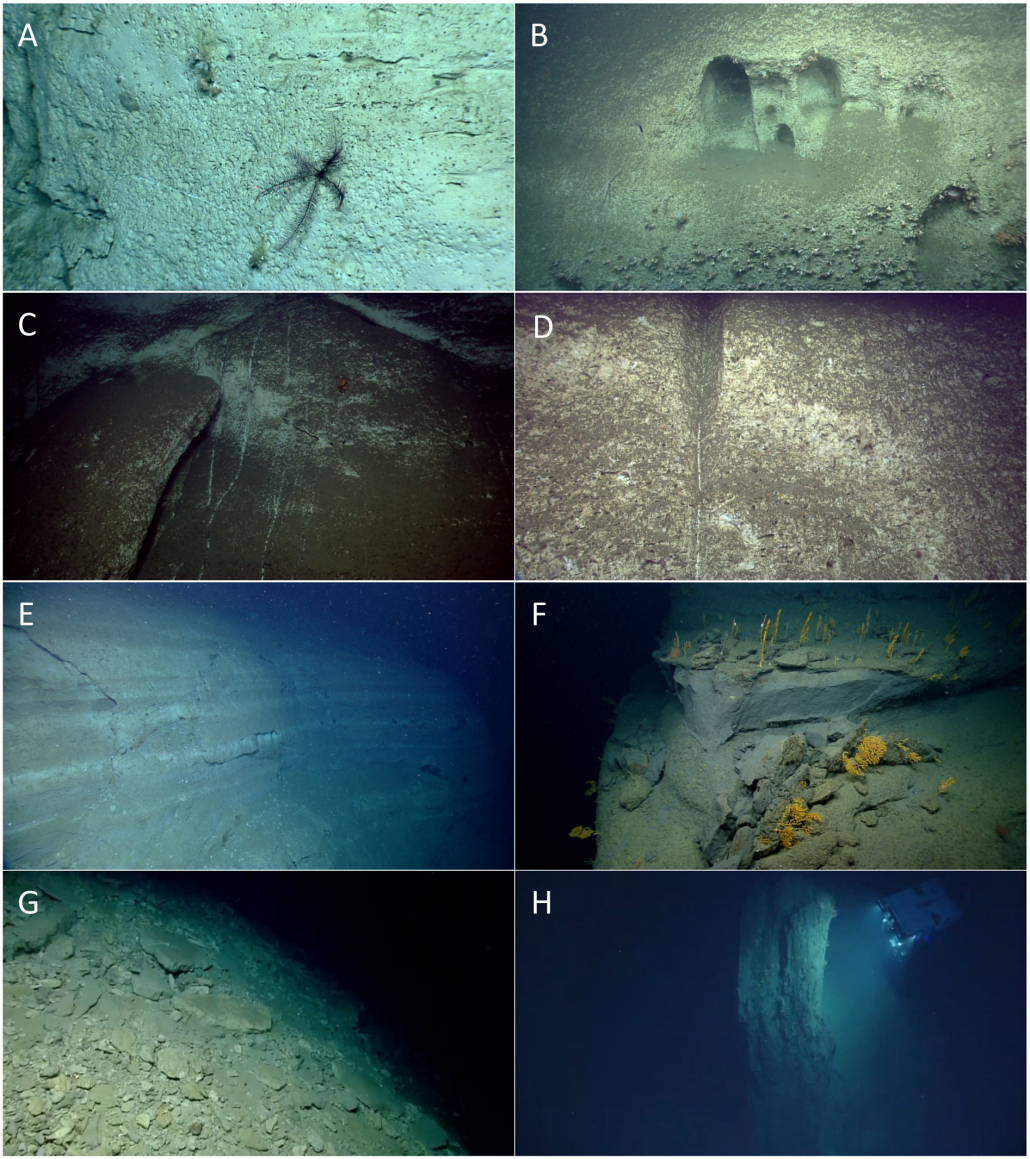
Examples of geology and geomorphology. (A) Vertical exposure of chalk in Atlantis Canyon showing biological and physical erosion morphologies (pits, burrows, horizontal striations). (B) Heavily eroded and partially colonized wall within Atlantis Canyon containing large caves. (C) Downslope abrasion marks along the wall of Alvin Canyon most likely from cascading sediment/water flows. (D) Transition from a well-developed erosional channel/chute to a linear abrasion mark on the wall of Alvin Canyon. (E) Spalling failure of a thin surficial layer of a layered mudstone exposure in Block Canyon. (F) "Recent" rockfall and spalling failure exposing a clean wall section within Oceanographer Canyon. (G) Debris apron at the base of a wall in Block Canyon. (H) ROV *D2* inspecting a large displaced block of layered mudstone, Heezen Canyon.

**Fig 3:**
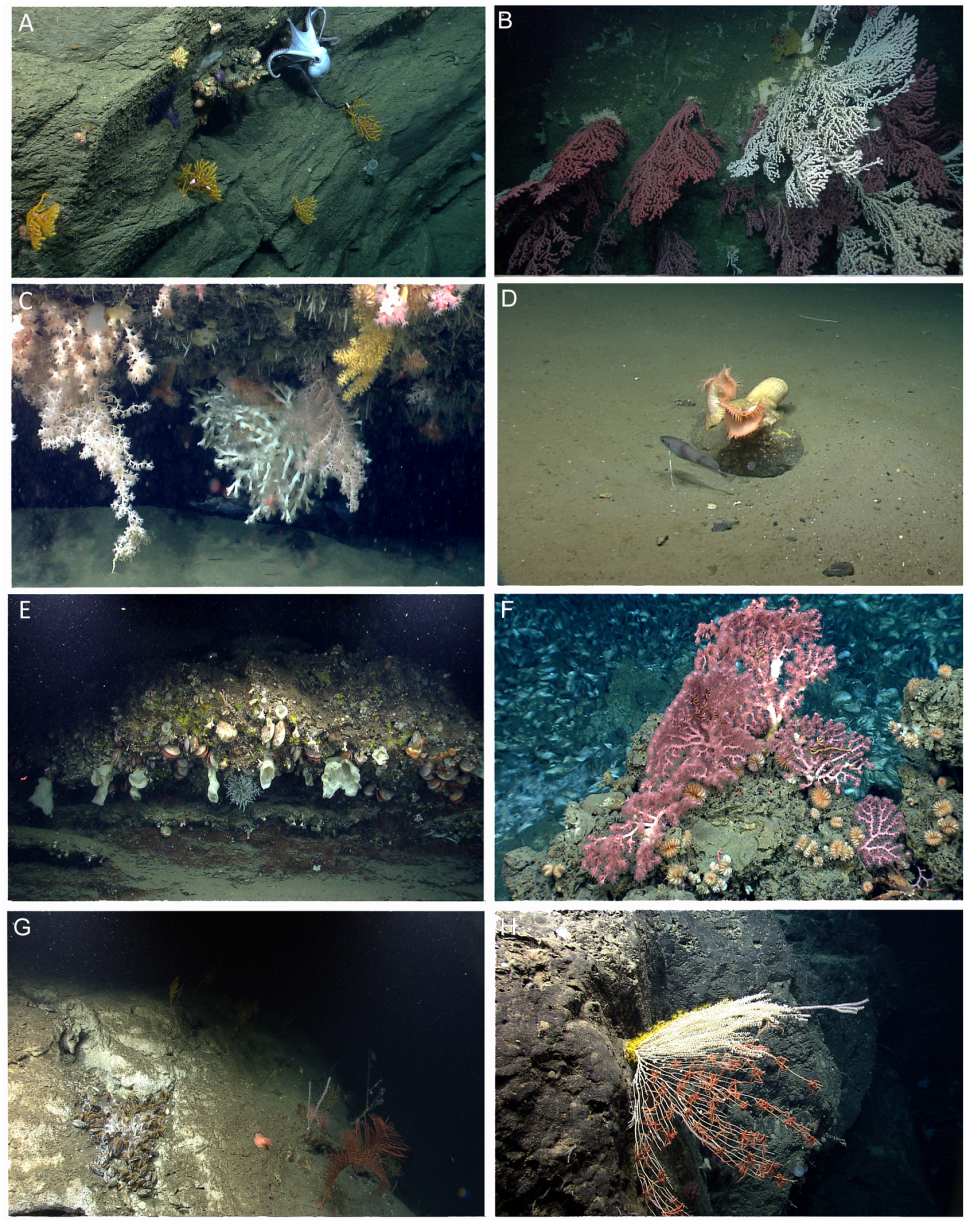
Benthic communities and habitats. (A) Corals and sponges colonizing a vertical wall in Hydrographer Canyon (1376 m) with the octopus *Muusoctopus johnsoniana*. (B) *Paragorgia arborea* colonizing the wall of Heezen Canyon (709 m). (C) *Lophelia pertusa* and additional sessile species growing on the underside of a ledge in Hydrographer Canyon (867 m). (D) An isolated dropstone in the Lydonia-Powell inter-canyon (501 m) with anemones (Hormathiidae) and *Phycis chesteri*. (E) A heavily colonized outcrop in the Nygren-Heezen inter-canyon area (793 m). (F) *Desmophyllum dianthus* and *P*.? *johnsoni* attached to authigenic carbonate at Veatch Seeps (1421 m). (G) *Bathymodiolus* sp. living on the wall of Nygren Canyon (1560 m). (H) Zoanthids and ophiuroid brittle stars covering a mostly dead primnoid octocoral on Mytilus Seamount (3057 m).

In the following sections, we provide an overview of the broad-scale habitat features observed during this expedition (Figs 2 and 3). These features are defined here as the major seafloor structures explored during ROV dives. These included eight shelf-breaching canyons (Block, Hydrographer, Alvin, Atlantis, Lydonia, Oceanographer, Nygren, and Heezen canyons, 580–2135 m), three slope-sourced canyons (Welker Canyon and two un-named canyons, 1018–1445 m), Mytilus Seamount (2592–3271), three cold seeps (1053–1484 m), two inter-canyon sites (494–824 m), and five sites on the open slope (555–2026 m), four of which were within submarine landslide scars (evacuation zones) (Table 1, S1 Table). We also provide notable observations of fauna associated with these broad-scale features.

### Broad-Scale Habitat Features

#### Shelf-breaching and Slope Canyons

Sixteen dives were conducted in shelf-breaching canyons and three dives were conducted in slope canyons (S1 Table). Shelf-breaching canyons connect the continental shelf to the abyssal plain whereas slope canyons are formed on the upper to lower continental slope and often do not extend onto the abyssal plain. Thick sequences of massive to thinly layered, eroded carbonate-rich/chalky mudstones and siltstones, sometimes intercalated with porcellaneous layers, were prominent along the canyon walls. Exposed faces of the chalk-rich layers were often etched and pitted, suggesting continuous biological, chemical, and physical erosion (S1 Table, Fig 2A and 2B). A similar sequence of carbonate rocks, Eocene-age silicious chalks and porcellanite was reported from the middle New Jersey continental slope at 1500 to 2500 m depth [60] and in Hendrickson Canyon in the Middle-Atlantic Bight [61].

Eleven dives were conducted in canyons along the Georges Bank section of the continental margin, from Hydrographer Canyon northward (Fig 1). Both silicilastic and carbonate-rich lithologic sequences were prevalent. Massive to thinly layered gray mudstones/siltstones, and white, pitted and striated, layered chalk/carbonate-rich rocks were observed. Outcrops in Hydrographer, Welker, Oceanographer, Lydonia and the deeper regions of Nygren and Heezen Canyons are most likely of Late Cretaceous to Eocene age; shallower areas in Nygren and Heezen Canyons are closer to Miocene-age [30, 63–65]. Heavy erosion, biological encrustation, and Fe-Mn oxide coating on almost all exposed rock surfaces in Nygren Canyon preclude identification of the canyon wall lithologies.

All canyons displayed evidence of sediment transport and progressive erosion by biological action, chemical solution, and physical abrasion, in addition to more episodic erosion by larger-scale slope failure/rock fall. Evidence of biological and chemical erosion was observed on all bedrock lithologies, except where surfaces had recently been exposed by failures. Erosion of these lithologies was primarily in the form of pitting/boring, horizontal striations, and development of large cavities (Fig 2A). Scour of the canyon walls by cascading sediment and/or water flows in the form of vertical scratches or wide chutes were observed. These features were most prominent in the chalk lithologies seen in Block, Atlantis, and Alvin Canyons (Fig 2C and 2D). While these scours were often sediment free, a number of the wider, better-developed chutes were dammed by coarse material causing sediment backfilling to occur. Evidence of sediment transport by bottom currents was not widely observed since the primary focus of the dives was on steep canyon walls rather than sedimented channels. However, sediment waves and sediment scour around the bases of larger debris blocks were observed in Heezen Canyon (dive 23).

Evidence of wall failure was observed in all canyons explored (Fig 2E–2H). Failure processes varied considerably from canyon to canyon, but lithological control on style and dimensions of the failures was apparent. Spalling of the rock face (Fig 2E and 2F), which resulted in removal of only a thin veneer of material from the surface of the rock walls, was commonly observed. At the base of the wall, small debris aprons were evident (Fig 2G). Large (>10 m) blocks (Fig 2H) were present in several canyons as either isolated features or were mixed with smaller-sized, displaced material within large debris aprons at the base of walls. Extensive debris aprons and talus slopes were present in Block, Alvin, and Atlantis Canyons. These features consisted primarily of tabular blocks, many meters wide, which were destabilized by erosion of the surrounding, weaker chalk layers (Fig 2).

Canyon walls and debris fields were colonized, in patches, by numerous species of sessile fauna, including corals, bivalves, anemones, and sponges (Fig 3A–3C). Scleractinians (e.g., *Desmophyllum dianthus*, *Solenosmilia variabilis*), octocorals (e.g., *Anthothela* spp., *P*.? *resedaeformis*) and bivalves (*Acesta* sp.) were often abundant under and around overhangs on the vertical canyon walls (Fig 3C). These same species also were often observed occurring in large patches on canyon walls that stretched for several 10s of meters. Notably, *L*. *pertusa* was documented in canyons at depths ranging from 733 to 1030 m (Fig 3C, S2 Table). Octopods (*Graneledone verrucosa*, *Muusoctopus johnsonianus*) and bobtail squids (*Rossia* sp.) were often observed along the canyon walls (Fig 3A). Although further quantification is required, areas within canyons appear to serve as nursery or spawning habitats for particular species (S2 Fig). For example, numerous individuals of *G*. *verrucosa* were observed along canyon walls guarding eggs. Bobtail squid eggs were observed within sponges growing on canyon walls. Catshark (Scyliorhinidae) egg cases were observed attached to corals. Skate (Rajiidae) egg cases were observed along and at the base of walls.

#### Open slope, Landslide Evacuation Zones, and Inter-canyon Areas

Five dives were conducted on the open slope; one near the shelf break (USGS2) and four in landslide evacuation zones (USGS1, 3–5). Two additional dives were conducted on the slope between canyons, termed inter-canyon areas (S1 Table). These regions were dominated by bioturbated, Pleistocene- to Recent-age unconsolidated sediments and glacial erratics. Pleistocene-age layered stratigraphy was exposed along the headwall scarp of a landslide evacuation zone immediately east of Block Canyon (USGS3, dive 1). Large debris blocks and scarps in other evacuation zones were draped with a layer of unconsolidated sediment at least 20–30 cm thick. Abundant glacial erratics, including rounded granitic boulders, were found on the seafloor and partially buried (dives 26 and 27) at the shallower (< 850 m) sites between Nygren-Heezen and Lydonia-Powell Canyons.

Corals and other sessile species were uncommon in most open-slope sites. However, some species of sea pens and bamboo corals that anchor in soft sediments were frequently observed during dive 12 on the channel floor of Veatch Canyon (S2 Table). One dive (dive 1) conducted on the open slope and one dive (dive 26) in an inter-canyon area traversed over several boulders and outcrops upon which corals, anemones, and sponges were attached (Fig 3E). Other areas had dropstones colonized by sessile fauna (Fig 3D). Mobile ophiuroids and holothurians were common at the deeper, sedimented open-slope sites (> 1600 m); whereas red crabs (*C*. *quinquedens)* and squat lobsters (*Munida* sp. 1) dominated the shallower, open-slope sites (<800 m). Areas where red crabs were present were heavily bioturbated with burrows. At USGS4 (dive 2), a high abundance (2.089 individuals 10 m^-2^) of red crabs was observed. Observations of at least 56 mating pairs and males actively caging females suggest that this area may be an important site for red crab reproduction. Red crab mating pairs were previously documented in nearby Veatch Canyon [68].

#### Cold Seeps

**Fig 4:**
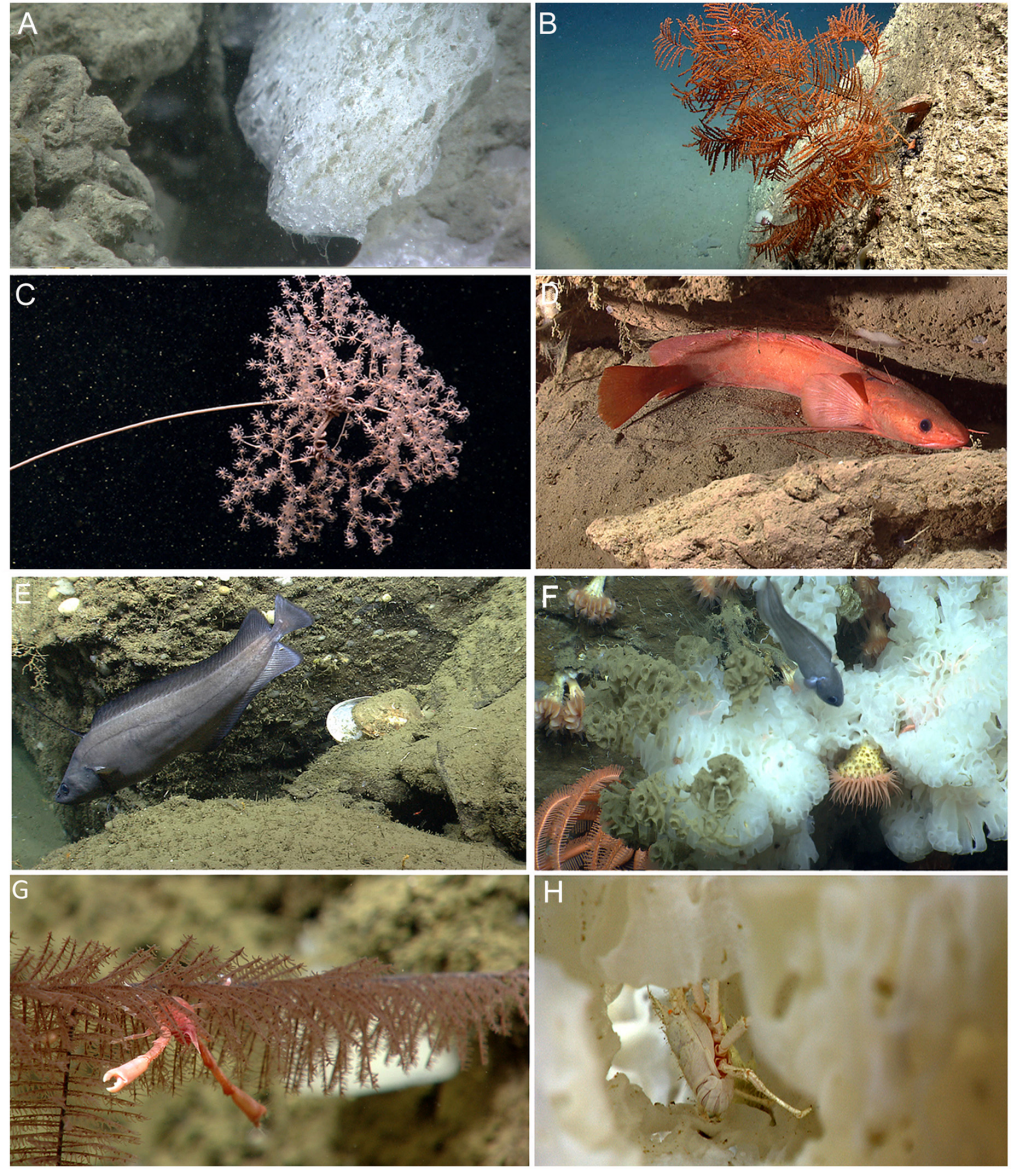
Notable observations and range extensions. (A) Methane gas hydrate observed at NE Seep 2 (1055 m). (B) *Telopathes magna* in Block Canyon (1345 m). (C) *Metallogorgia melanotrichos* in Atlantis Canyon (1755 m). (D) *Gaidropsarus argentatus* under a ledge in Block Canyon (1008 m). (E) *Lepidion guentheri* in Oceanographer Canyon (995 m). (F) *Guttigadus latifrons* in Heezen Canyon (1645 m). (G) *Uroptychus* sp. inhabiting *Parantipathes* sp. in Oceanographer Canyon (1079 m). (H) *Munidopsis* spp. inhabiting a hexactinellid sponge in Atlantis Canyon (1744 m).

Three dives (dives 3, 4, 13) were conducted at inter-canyon sites to ground truth multibeam backscatter data that showed plumes of bubbles rising from the seafloor (see [46]). These dives documented living chemosynthetic communities at depths ranging from 1053 to 1484 m (Fig 1, S1 Table). Observations of gas hydrates (Fig 4A), scattered empty mussel shells, live mussels, carbonate rocks, microbial mats, and dark patches of apparent anoxic sediment proved that these areas were either sites of active seepage or historical seep sites. Bubbles were observed (Veatch Seeps, NE Seep 2) escaping from the seafloor as was predicted from the multibeam backscatter data. Methane hydrate was also documented at NE Seep 2 and 3 (also see [46], for observations and supplementary video clips). Seafloor gas hydrate was previously documented on the U.S. Atlantic margin at the Blake Ridge seep, off the coast of South Carolina at 2000 m depth [69]. The gas hydrates observed on the NEUS expedition generally occur beneath small overhangs, the same setting in which the Blake Ridge seep seafloor hydrate was found. In these settings, gas bubbles emitted from the seafloor supersaturate waters beneath the overhang with methane, provoking the formation of porous gas hydrate around the bubbles, which then combine to form a gas hydrate mass. The NEUS seafloor gas hydrates occur at pressure and temperature conditions that are within the hydrate stability field. This seafloor hydrate population is distinct from the upper slope gas hydrates (~550 m water depth) that may feed a large proportion of the seeps described by Skarke et al. [46].

A living chemosynthetic community was also discovered (dive 22) along the west wall of Nygren Canyon at a depth of 1560 m (Fig 3G). In contrast to the other seep sites investigated, water column backscatter data did not suggest the presence of a seep community in Nygren.

*Bathymodiolus* spp. was the dominant species observed in each of the cold seep sites. Small patches and large, expansive (>100 m in length) beds consisting of individuals of different size classes were observed. Interestingly, 1000s of small individuals, indicating ongoing and/or recent recruitment to the area, were observed at Veatch seeps (dive 13). At NE Seep 3, different sized patches of live and dead mussels were commonly encountered. Patchy distribution of mussel beds and the size variability of individual mussels within a bed may be indicative of methane flow that varies both temporally and spatially [69–71]. In contrast to cold seeps in the Gulf of Mexico [48], the Mediterranean [72] and off West Africa [73], the NEUS seep sites lacked vestimentiferan tubeworms. Absence of vestimentiferans and vesicomyid clams may signify either lack of hydrogen sulfide and/or an insufficient supply of larvae from source populations to maintain a local population [74–76], thus suggesting that these NEUS seeps may be functionally different from those in other regions.

Species associated with the NEUS seep mussel beds included endemic gastropods and shrimps (*Alvinocaris* spp.) as well as background fauna (e.g., *Antimora rostrata*, *Synaphobranchus* sp., *Echinus* sp., *C*. *quinquedens)* not specific to seep habitats (S4 Table). Additionally, octocorals (*Paragorgia*? *johnsoni*,? *Hemicorallium* sp.) and scleractinian cup corals (*D*. *dianthus)* were attached to authigenic carbonates at Veatch seeps and the canyon wall adjacent to the seep community in Nygren Canyon (Fig 3F and 3G).

#### Mytilus Seamount

Mytilus Seamount is a guyot, with the cap composed of recrystallized limestone dominated by fossil Melobesiacean algae [77, 78]. On the NEUS expedition, the north and south sides of this seamount were explored during two dives (dives 20, 21) at depths ranging from 2592 to 3271 m. Both sides of the seamount were characterized by gradually- to steeply-sloping pillars that were smooth in texture and interspersed with ledges covered with sediment. Piles of manganese-coated rocks as well as material that resembled carbonate debris were observed, indicating some slope instability and possible erosion. Pillow lava was also observed, which is consistent with the volcanic origin of this seamount [79]. Previous submersible investigations noted that certain outcrops on Mytilus Seamount appeared to be igneous in nature; however, of the samples collected, none were basalts [78]. Compared with the transect on the north side, the transect up the south flank of the seamount appeared to have expansive areas of soft-sediments with scattered cobbles.

A diverse assemblage of deep-sea taxa was observed on the seamount (S2, S3 and S4 Tables), including numerous hexactinellid sponges, demosponges, bryozoans, antipatharians, and octocorals. Notable was the discrete zonation of epifauna living on a large primnoid octocoral on the southern wall. Ophiuroids, barnacles, and zoanthids covered different sections of this mostly dead coral (Fig 3H). Echinoderms (all classes represented) were the most common and diverse members of the mobile megafaunal assemblage observed.

### Benthic Assemblage Structure

#### Corals

**Fig 5:**
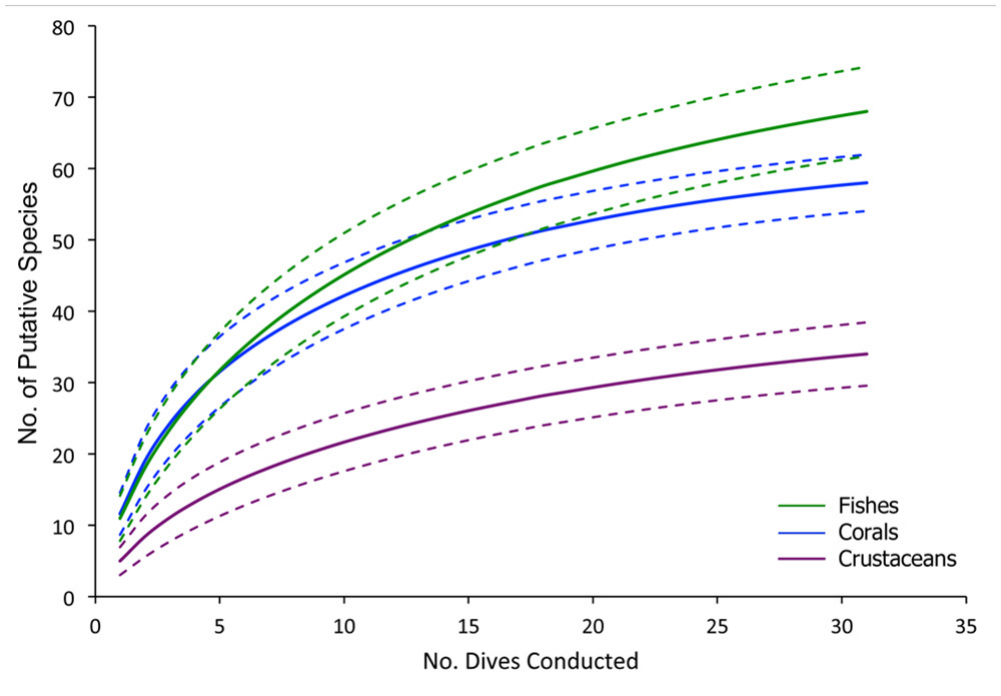
Cumulative number of putative species (solid line) by taxon observed across all dives conducted. 95% confidence intervals are indicated (dotted lines).

At least 58 coral taxa representing 20 families from four orders were documented along the NEUS continental margin (S2 Table). The species accumulation curve appeared to approach an asymptote, suggesting that coral genera were well documented throughout the region (Fig 5). However, additional species will likely be added to the regional species list with further exploration and targeted collections. We emphasize and recognize that specimen collections are critical to corroborate identifications from video imagery. Many taxa cannot be identified to species level from images alone. Furthermore, there is a high likelihood of discovering new, cryptic, and/or incipient species in these underexplored habitats [80–82]. Genetic analyses combined with microscopic examination of morphological characters are necessary to confirm identifications.

Despite using a conservative approach to coral identification, the species list compiled during the present study illustrates that corals are much more diverse in the NEUS region (depths > 200 m) than previously recognized. Published annotated checklists for the region (including Gulf of Maine, Georges Bank to Cape Hatteras, NC, and U.S. EEZ Northeast Seamounts) report 25 species of alcyonacean octocorals [45], 17 species of pennatulacean octocorals [51], 16 species of scleractinians [83], and three species of antipatharians [51, 84]. Of the total 58 coral taxa we documented, at least 24 species (identified to the lowest possible taxon) were not previously recorded for the coral fauna in the region. This suggests gamma diversity in the NEUS region is potentially higher than in other regions of the western North Atlantic, given that many taxa were identified only to genus in the present study. For example, approximately 30 species were documented within similar depth ranges off Newfoundland and in the Labrador Sea [85, 86], although differences in observational effort and methods need to be acknowledged. Regardless, the high gamma diversity along the NEUS slope is likely due to high habitat heterogeneity (e.g., canyons, landslide scars, seamounts) in the region across a broad depth range (~200 to 3500 m). Additionally, because submarine canyons can channel organic matter [8] and thus enhance food supply, the NEUS canyons may contain higher biodiversity and density of fauna compared with other areas along continental margins [11, 21, 87].

Based on our observations, geographic range extensions are apparent for several coral species, although taxonomic voucher specimens are necessary to confirm these identifications. Of note, the black coral *Telopathes magna* (Fig 4B) was previously known only from the continental slope off Nova Scotia and the New England Seamount Chain [84]. Based on our knowledge of colony morphology, we observed this species during 11 dives. The octocoral *Metallogorgia melanotrichos* was observed (Fig 4C) during one dive in Atlantis Canyon. Previously, this species had not been documented on the Atlantic continental margin of the U.S. north of the Gulf of Mexico, although it occurs offshore on the New England and Corner Rise Seamounts [41, 88, 89], in the Pacific [88], the Mediterranean [89], the Gulf of Mexico (A. Quattrini, T. Shank, pers. observ.), on the Bahamas Escarpment, and in Little Abaco Canyon (S.C. France, pers. observ.).

**Fig 6:**
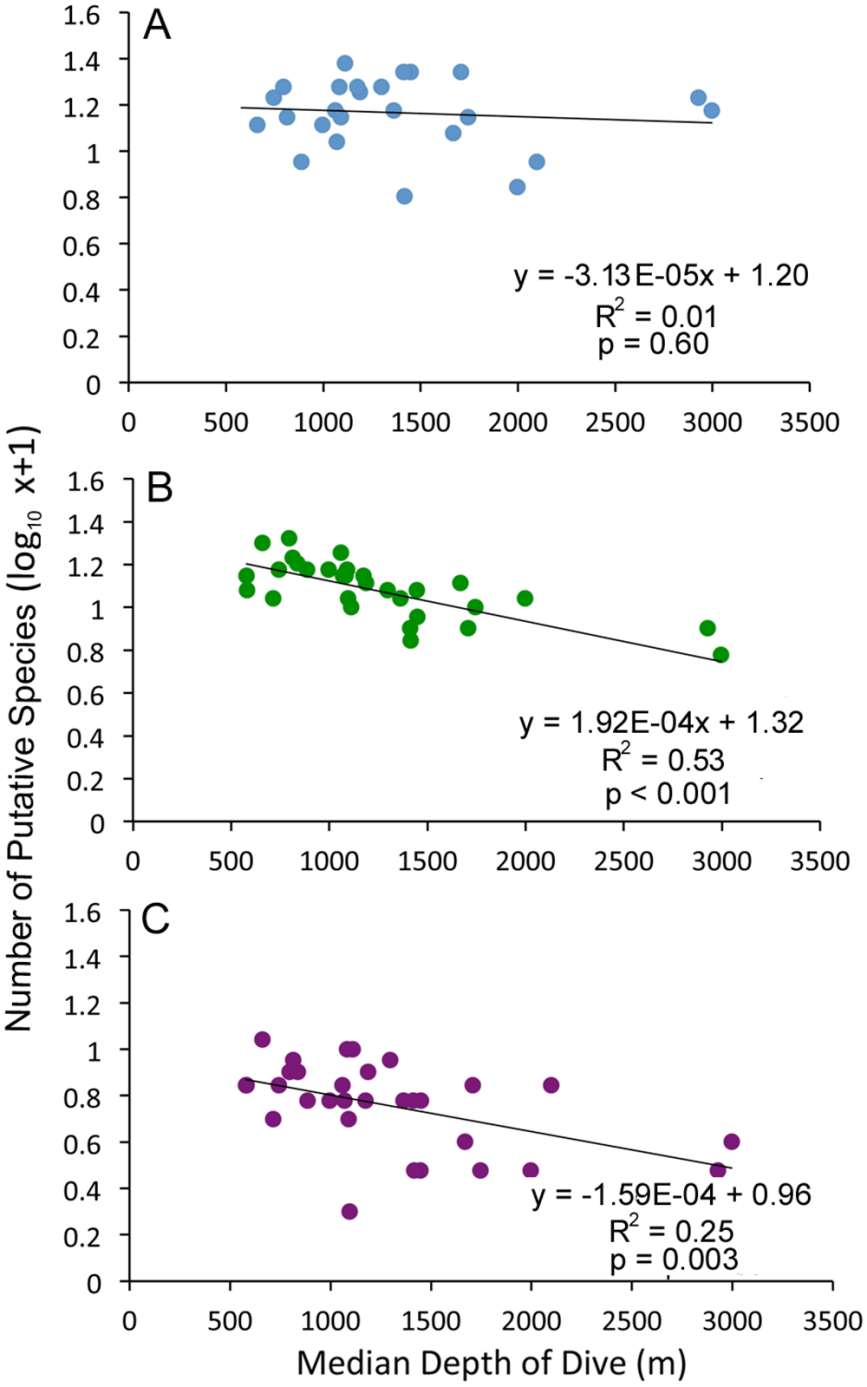
Species richness by depth. (A) corals. (B) fishes. (C) crustaceans. Best-fit linear trend lines are included.

**Fig 7:**
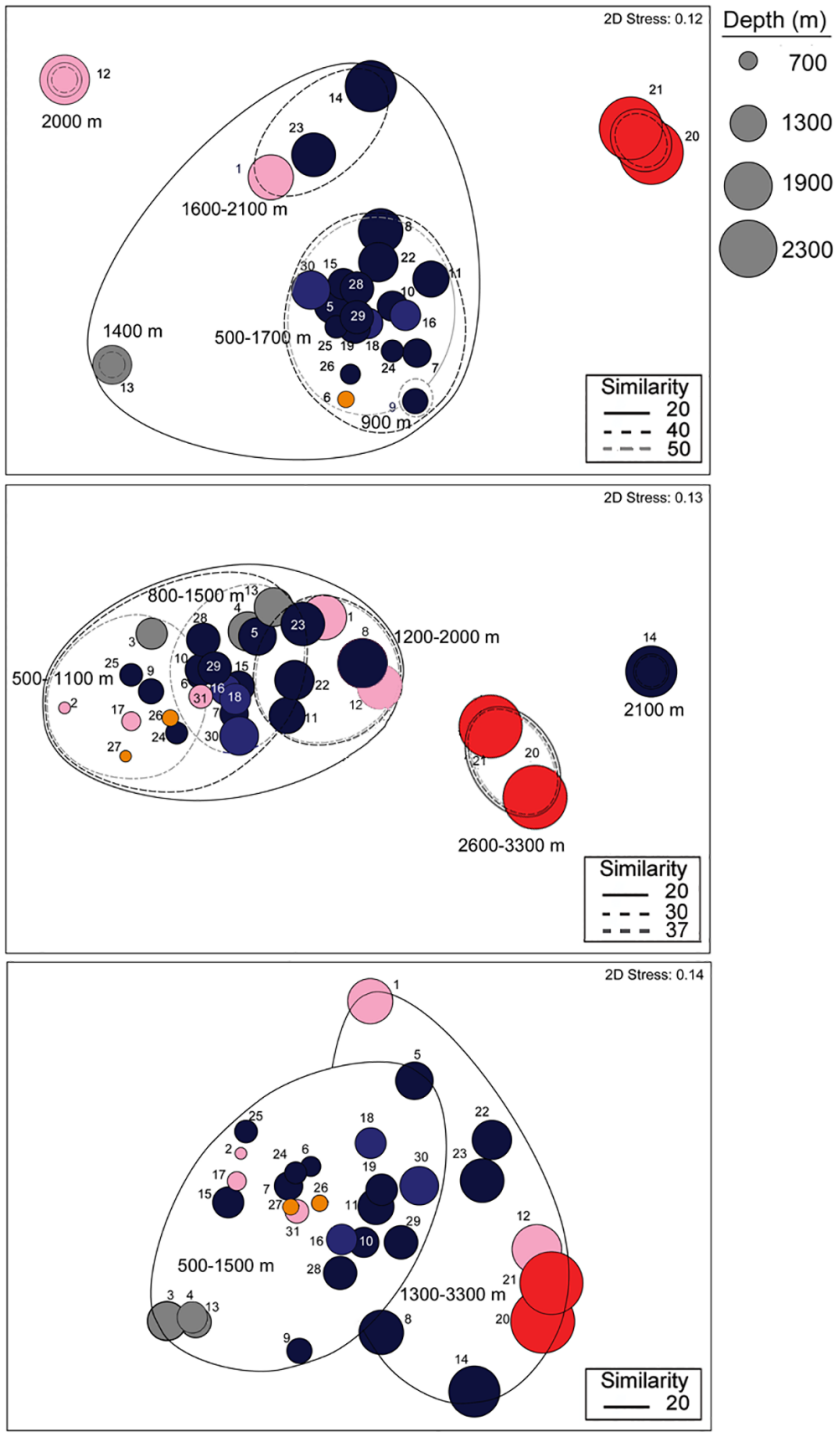
Non-metric multi-dimensional scaling plots based on Sorensen's Index of Similarity. (A) corals. (B) fishes. (C) crustaceans. Significantly (SIMPROF, p<0.05) dissimilar assemblage clusters are noted. Dive numbers are noted. Circle size denotes median depth of dive. Pink = Open Slope/Landslide Scar, Orange = Inter-canyon Slope, Gray = Cold Seep, Blue = Slope Canyon, Dark Blue = Shelf-breaching Canyon, Red = Seamount.

Depth was a significant factor influencing coral assemblages (DistLM marginal test, p = 0.001, Table 2). Although coral species richness did not change significantly (linear regression, R^2^ = 0.02, p>0.05) with depth over the depth range explored (494 to 3271 m, Fig 6A) species composition of corals changed at approximately 1600 to 1700 m (Fig 7A). Species composition in canyons and other environments containing hard substrates were significantly dissimilar across this depth boundary (60%, SIMPROF, p<0.05, Fig 7A). Scleractinians and the octocorals *Anthothela* spp., *Keratoisis* sp. 1, and *Paragorgia arborea*, occurred at depths <1700 m, whereas chrysogorgiids and sea pens were more common at depths >1700 m (SIMPER). Additionally, the coral assemblage observed on Mytilus Seamount (> 2600 m) was significantly dissimilar (80%, SIMPROF p<0.05) from coral assemblages at other sites. Differences in species composition between Mytilus Seamount and other sites were primarily driven by the presence/absence of numerous species. *Chrysogorgia* spp., *Convexella*? *jungerseni*, *Corallium*? *bathyrubrum*, *Paranarella*? *watlingi*, and *Paragorgia/Sibogagorgia* sp. 1 were observed on Mytilus Seamount, whereas *Acanthogorgia* spp., *Anthothela* spp., *Clavularia*? *rudis*, *P*. *arborea*, and *Paramuricea* spp. were not seen on Mytilus Seamount, but occurred at other sites (SIMPER). Notably, no scleractinian corals were observed at Mytilus Seamount, perhaps because these deeper depths (2600 to 3200 m) are beyond the bathymetric limits of scleractinian species occurring in the area.

Broad-scale habitat feature was another important factor that influenced coral assemblages (DistLM marginal test, p = 0.005, Table 2). Coral assemblages at Veatch seeps and USGS2 were significantly dissimilar (70–80%) from all other sites (SIMPROF, p<0.05, Fig 7A). Additionally, low alpha diversity was observed at Veatch Seeps and USGS2 compared to the other sites investigated. These differences are related to habitat suitability, i.e., the underlying substrate type and availability of hard substrata. Five species of corals (S2 Table) were observed attached to carbonate blocks surrounding the live mussel bed at Veatch Seeps. No hard substrates were observed at USGS2, but four species of sea pens and two bamboo corals were observed anchored into soft sediment. No octocorals were observed during six dives (dives 2–4, 17, 27, and 31) that were conducted over soft sediment on inter-canyon and open slopes.

In combination, the factors of depth, habitat, salinity and DO explained 71% of the total variation observed in coral assemblage structure (BEST model; AIC = 135.85). Depth (and the factors that co-vary with depth including salinity) has previously been shown to be important in structuring deep-sea coral assemblages in other regions, including the continental slope off Newfoundland [86], Hawaii [66], and in the Gulf of Mexico [82]. Coupled ecological and evolutionary processes influence species composition and diversity of deep-sea coral assemblages over an environmental gradient of depth. Specifically, depth-related mechanisms influencing coral distributions include, but are not limited to, restricted gene flow across water mass boundaries, potential adaptation to environmental factors (e.g., pressure and temperature, [90]), historical colonization events [91], and *in situ* diversification [88]. Additionally, the distribution and suitability of habitat features including substrate type (e.g., soft sediment, hardbottom) and topographic variables (e.g., rugosity, slope) are known to influence coral assemblage structure [66, 86]. Although we found no significant differences between assemblages in slope and shelf-breaching canyons, lower diversity and a different faunal assemblage were noted at cold seeps and open-slope sites that lacked hard substrates.

**Fig 8:**
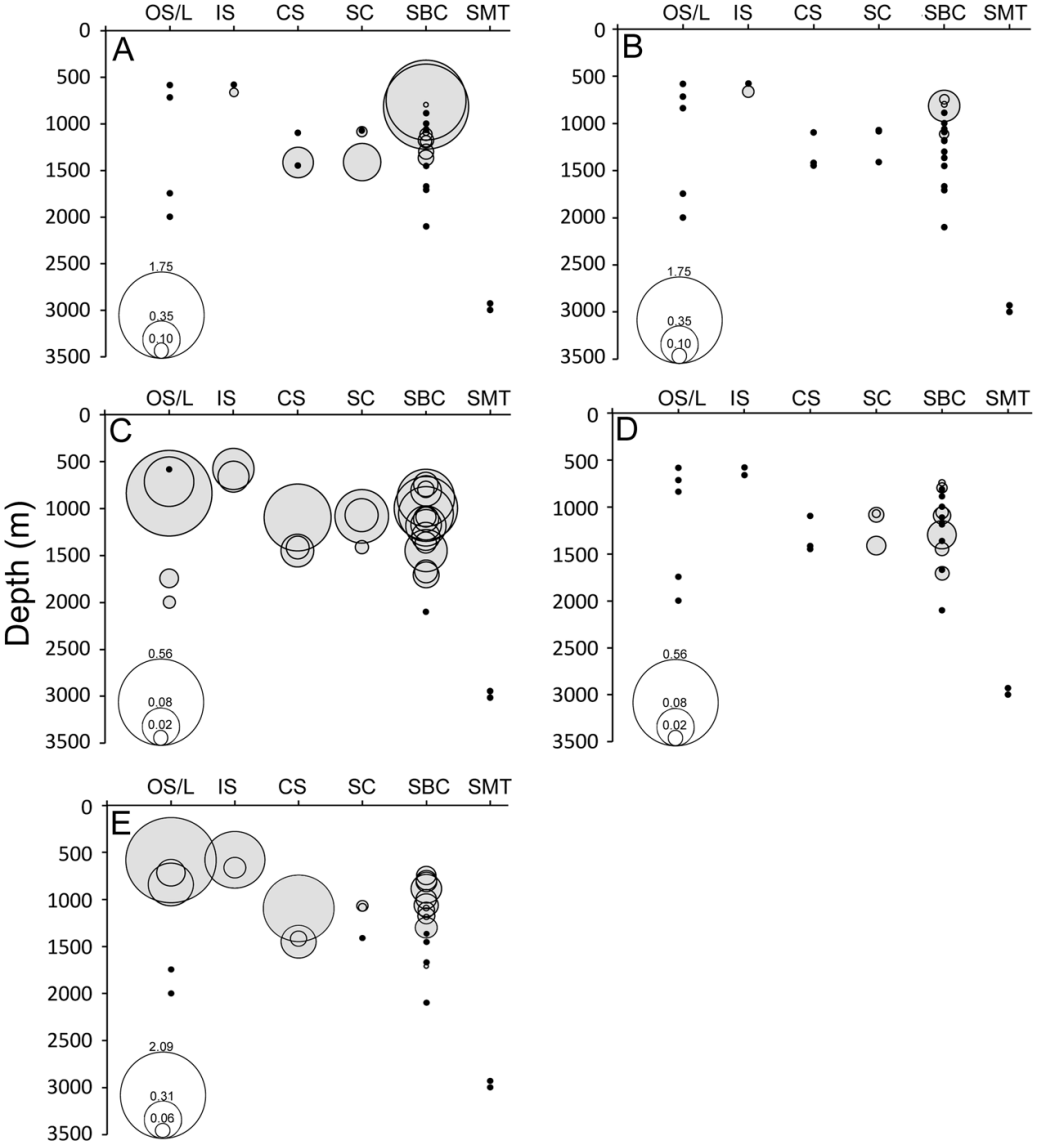
Abundance estimates of dominant species in the region. (A) *Paragorgia* spp. (B) *Primnoa*? *resedaeformis*. (C) *Synaphobranchus* spp. (D) *Neocyttus helgae*. (E) *Chaceon quinquedens*. Circle size corresponds to number observed 10 m^-2^ as shown in key inset at lower left of each graph. Black symbols denote dives in which no species were observed. OS/L = Open Slope/Landslide Scar, IS = Inter-canyon Slope, CS = Cold Seep, SC = Slope Canyon, SBC = Shelf-breaching Canyon, SMT = Seamount.

Coral abundance estimates ranged from 0.005 to 1.756 colonies 10 m^-2^ for *Paragorgia* spp. and 0.007 to 0.233 colonies 10 m^-2^ for *P*.? *resedaeformis*. These estimates are similar to abundances of *P*. *resedaeformis* and *P*. *arborea* measured off Nova Scotia [59]. However, in contrast to the present study, *Primnoa* was more abundant than *Paragorgia* in that region. Abundances of both species in the NEUS differed among habitats and declined with increasing depth (Fig 8A and 8B); a pattern also reported by Watanabe et al. [59]. The highest abundances recorded in this study of both *Paragorgia* spp. (1.548 to 1.756 colonies 10 m^-2^) and *P*.? *resedaeformis* (0.233 colonies 10 m^-2^) were found in shelf-breaching canyons at depths of approximately 800 m. These estimates, calculated across an entire dive transect, do not reveal the high variability in abundance and the patchy distribution seen for many of the corals over the course of a single dive or across sites at similar depths. Although a more rigorous sampling design needs to be incorporated to accurately compare abundance patterns across depths and habitats, for many of the coral taxa observed, variation in abundance across habitats does not appear to be related only to depth (see also [59]). For example, both the highest and lowest abundances of *P*. *arborea* were found at approximately 800 m in Hydrographer and Nygren Canyons, respectively. Other factors, including the abundances of other sessile species, the stability of canyon walls, current regimes, and/or food availability, could be driving the variation in coral abundances, and warrant further investigation throughout the region.

#### Demersal Fishes

A total of 69 demersal fish species representing 30 families were documented along the NEUS continental margin (S3 Table). The species accumulation curve did not quite reach an asymptote, suggesting more species will likely be documented with further exploration (Fig 5). Combining species checklists from [92–94], the estimated number of fish species in the NEUS region is 630. Of these 630 species, approximately 153 are demersal and benthopelagic species that occur in this region within similar depth ranges as surveyed in this study (J. Moore, unpubl. data). Thus during the NEUS expedition, the ROV *D2* documented only 45% of the demersal fishes known in the region. It is likely that the ROV did not adequately document certain species that either avoid (e.g., sharks, chimaeras) underwater vehicles [95, 96], are too small and cryptic to be observed using video, or occur at such low densities that encounter rates with survey vehicles are exceedingly low. Combining methods of submersibles, surface-deployed gear (see [97]), and even museum collections [92] are ideal in order to provide voucher specimens and a more comprehensive, regional assessment of fishes.

The ROV surveys in the present study, however, did document the numerically dominant fish species found in the region (e.g., *Antimora rostrata*, *Glyptocephalus cynoglossus*, *Nezumia bairdii*, *Phycis chesteri*, and *Synaphobranchus* spp.; [98]); capture images of species rarely observed *in situ* (e.g., *Cottunculus thomsonii*, *Harriotta raleighana*, and *Paraliparis copei*); and document at least three species not previously recorded from the area. *Gaidropsarus argentatus* was previously known from the Grand Banks to Greenland [99] with one specimen collected on the New England Seamount Chain [92]. We noted several occurrences of this species during seven dives along the continental slope, suggesting that this species may be more common than previously realized (Fig 4D). Two individuals of *Lepidion guentheri* were observed in Oceanographer Canyon and one individual was observed in Nygren Canyon (Fig 4E). This species was previously known only from the eastern Atlantic [100]. *Guttigadus latifrons*, a species previously known from the Mediterranean Sea and northeastern Atlantic [101] and New England Seamount Chain (J. Moore, pers. observ.) was observed in Heezen Canyon (Fig 4F). The association of these fishes with rugged topography and hard bottom habitat may have precluded them from being discovered in this region, as most work to date has been based on trawl surveys [92].

*Neocyttus helgae* was observed (n = 39) on ten separate dives in Block, Alvin, Welker, Hydrographer and Nygren Canyons as well as two un-named canyons, just west of Hydrographer and Block. This species was considered to be distributed across the northeast Atlantic region, with a range as far west as the Corner Rise and New England Seamounts [96]. It was suggested that *N*. *helgae* used seamounts as stepping-stones to produce this distributional pattern (with depth bounds of 915 to 1829 m [94]). An observation of a single individual in Lydonia Canyon (1469 m), based on a photographic record from a single DSV *Alvin* dive in 1982, was also reported [96]; the authors suggested this was possibly a failed colonization event. Subsequent to that study, *N*. *helgae* was reported in three canyons south of Newfoundland (from approximately 800–1500 m) [102] and recently in Norfolk Canyon [97]. Whether or not occurrences of *N*. *helgae* represent a previously unknown historic range due to under-sampling in canyon habitats or is evidence of a recent range expansion remains to be considered.

Depth was a significant factor affecting assemblage structure of demersal fishes (DistLM marginal test, p = 0.001, Table 2). Species richness of demersal fishes declined with increasing depth (linear regression, R^2^ = 0.53, p<0.001, Fig 6B). A decline in species richness with depth is commonly observed in this depth range [98, 103–105], and has been linked to food availability [103]. The species composition of demersal fishes also changed with depth; however the change in assemblage structure was more gradual rather than an abrupt cline or a complete species turnover. Three of the five clusters that were significantly dissimilar (60–80%) from one another did not correspond to any abrupt breaks in species distribution within the 500 to 2000 m depth range (Fig 7B). Several species commonly observed across a large portion of this depth range included: *A*. *rostrata*, *G*. *cynoglossus*, *N*. *bairdii*, and *Synaphobranchus* spp. (SIMPER). Therefore, the observed differences in species composition of fish assemblages are due to either the appearance or disappearance of other species along the depth gradient. Species common at deeper depths included: *Aldrovandia affinis*, *Halosauropsis macrochir*, and *Luciobrotula corethromycter*. Species more common in shallower depths included: *P*. *chesteri*, *Helicolenus dactylopterus*, and *Sebastes mentella* (SIMPER).

Marked changes in fish assemblage structure occurred at depths >2000 m. Low species richness (3 species; *Bathysaurus mollis*, an unidentified ophidiiform, and *Coryphaenoides* cf. *carapinus*) was observed in Block Canyon at 2062 to 2135 m depth. Species richness was also low (7 species) at Mytilus Seamount, and included *B*. *mollis*, *Chaunacops roseus*, *Coryphaenoides armatus*, Synaphobranchidae sp. 1, and an unidentified ophidiid (sp. 3). Also at Mytilus Seamount (>2600 m), species composition was 80% dissimilar from other sites (SIMPROF, p<0.05). Species present on Mytilus Seamount are not endemic to seamounts [92], but they do appear to be more common in deeper depths [92, 106].

Broad-scale habitat features also influenced assemblages of demersal fishes (DistLM marginal test, p = 0.007, Table 2). Although no significant groupings (SIMPROF) were evident in the MDS plot (Fig 7B), assemblages were similar at several sites in either canyon or open-slope environments. Several species appeared to be tightly associated with canyon walls and/or were found hiding within burrows and undercuts in the walls. These species included? *Grammonus* sp., *G*. *latifrons*, *L*. *guentheri*, and *N*. *helgae*. *Gaidropsarus* spp. and *L*. *corethromycter* were also commonly documented along canyon walls, but these species also were observed under ledges in other hardbottom environments. Similarly, various species such as *Bathysaurus ferox*, *Bathypterois grallator*, *Lophius americanus*, *Rajella bigelowi*, an unidentified rajiid skate, and *Chlorophthalmus agassizi* were present only at soft-sediment, open-slope sites. *Chlorophthalmus agassizi*, in particular, is a common inhabitant of soft substrate and open-slope habitats [40].

A combination of all environmental factors examined in this study explained 71% of the variation in assemblage structure (DistLM, BEST, AIC = 178.44). Distance covered by the ROV was retained in the BEST model; however, distance only added 2% of explained variation and was not significant when considered alone (DistLM marginal test, p = 0.282). Habitat and depth (and the co-factor salinity) accounted for most of the variation in the model. The assemblage structure of fishes over soft-substrate habitats has been shown to differ from those associated with more complex structures, such as deep coral and hardbottom reefs [40, 107]. Similarly, changes in assemblage structure of fishes commonly occur along a depth gradient [17, 97, 108–110]. In the present study, many species were common across a broad depth range and the changes in assemblage structure with depth appeared to be largely a consequence of declining alpha diversity. Rate of species turnover and the particular depths where turnover occurs likely differs among regions due to the overlying water mass characteristics, habitat heterogeneity, and food quantity/quality within regions [111]

Interestingly, coral species richness was also an important variable in explaining assemblage structure (DistLM, BEST, AIC = 178.44). Corals may increase local diversity of demersal fishes as some species use corals as refugia or a central base for foraging [94] or as spawning habitat (e.g., catsharks [44] liparids [97]). Coral species richness may also be an indicator of an additional, yet unmeasured, environmental variable that influences the distribution and abundance of fishes. For example, areas that host more coral species may contain increased amounts of organic matter and thus food resources. Additionally, the occurrence of specific relationships between corals and invertebrates [35, 41, 42] may increase the diversity and availability of prey items to fishes.

Abundances estimated for *Synaphobranchus* spp. and *N*. *helgae* peaked at mid-depths (Fig 8C and 8D) and further revealed differences in habitat-use patterns. *Neocyttus helgae* was only observed in canyon habitats, with highest abundances at depths of approximately 1200–1500 m (Fig 8D). This species was not found within other structurally complex habitats (e.g., cold seeps) surveyed in similar depth ranges. Occurrences in canyon habitats could be driven by presence of refugia or because of enhanced food supply [96]. Abundances were similar between slope (0.004 to 0.026 individuals 10 m^-2^) and shelf-breaching (0.003 to 0.060 individuals 10 m^-2^) canyons (Fig 8D). In comparison, *Synaphobranchus* spp. was a habitat generalist [112]. *Synaphobranchus* spp. was found across most depths and habitats surveyed (except Mytilus Seamount); although more than one species may be present in the region [92]. This generalist habitat strategy was also observed in Norfolk and Baltimore Canyons and adjacent cold seeps in a recent study [97]. *Synaphobranchus* spp. was most abundant (mean 0.197 ± 0.256 S.D individuals 10 m ^-2^) in open slope/landslide scar areas, with the highest abundance (0.563 individuals 10 m^-2^) estimated during at USGS5 (dive 31). Abundances of *Synaphobranchus* spp. also peaked at mid-depths of approximately 700–1200 m. A peak in abundance at similar depths was reported previously for *S*. *kaupii* [113].

#### Crustaceans

A total of 34 megafaunal decapod crustacean morphospecies were documented along the NEUS continental margin (S4 Table). Of these crustaceans, shrimps were the dominant group (46%) followed by galatheiod squat lobsters (18%) and pagurid hermit crabs (12%). Brachyurans, lithodids, and chirostylids represented 24% of the crustacean fauna. The species accumulation curve appeared to reach an asymptote indicating that crustacean morphotypes/genera were well documented (Fig 5). Although the high-definition cameras on the ROV allowed for numerous zooms, a portion of the crustacean fauna was likely not observed as many species are cryptic, inhabit burrows and crevices, and are often found intimately associated with corals and/or sponges (Fig 4G and 4H). Additionally, a large portion of the unexplained variation (33%, Table 2) in crustacean assemblage structure may be due to insufficient taxonomic resolution at the species level. For example, the identification of many of the individuals in the genus *Munidopsis* could not be resolved to species or a distinct morphotype. These individuals often occurred deep within scleractinian coral bushes, obscuring many diagnostic characters. Thus, *Munidopsis* spp. was not included in PRIMER analyses. Targeted collections are needed to identify the morphotypes to species-level since many diagnostic characters cannot be evaluated from images alone.

As with other taxonomic groups, depth was a significant factor affecting assemblage structure of crustaceans (DistLM marginal test, p = 0.001,Table 2). Species richness of crustaceans declined with increasing depth (linear regression, R^2^ = 0.25, p = 0.003, Fig 6C). Crustacean assemblage structure was significantly dissimilar (80%, SIMPROF, p<0.05) at a boundary of around 1300 to 1500 m (Fig 7C). Species composition at depths <1500 m were dominated by *C*. *quinquedens*, banded Shrimp sp. 2, *Munidopsis* sp. 1, *Munida* sp. 1, and *Munida* sp. 2. In contrast, Paguridae sp. 3, Shrimp sp. 3, and *Munidopsis* sp. 3 were common at depths >1300 m (SIMPER).

Broad-scale habitat features also influenced assemblage structure of crustaceans (DistLM marginal test, p = 0.001, Table 2). Although assemblages at open-slope sites did not form a tight grouping, several species (e.g., squat lobsters) that are intimately associated with corals were notably absent from open-slope sites (S4 Table). Additionally, *Paralomis* cf. *bouvieri* was only observed within canyon environments. The crustacean assemblage at USGS3 (dive 1) was also highly dissimilar (70%) from other sites in the same depth range. Low alpha diversity (two species) was recorded during this dive along a landslide scar on the open slope (S4 Table). Low species richness (1–2 species) of crustaceans also occurred at each of the cold seep sites (S4 Table). Assemblages were 70% similar among the cold seep sites, although not significantly so (SIMPROF, p>0.05, Fig 7C). *Alvinocaris* sp., a shrimp endemic to chemosynthetic communities, was only observed at the cold seep sites.

The BEST model indicated that salinity, depth, coral species richness, and broad-scale habitat features were the best combination of variables that explained the observed structure of the crustacean assemblage (DistLM, BEST, AIC = 178.49). Similar to fishes and corals, bathymetry was important in shaping deep-sea crustacean assemblages. A decline in species richness with depth and a change in species composition were reported on the middle to lower slope in other areas of the western North Atlantic, including off New England [98] and in the Gulf of Mexico [114, 115]. Our analyses also revealed the added importance of broad-scale habitats and coral species richness in shaping crustacean assemblage structure. The diversity of corals in canyon environments influences the differences observed between the canyon and open-slope habitat features. Several species of crustaceans, particularly galatheoid and chirostylid squat lobsters, are intimately associated with corals. Only *Munida* spp. were commonly and consistently observed on soft sediments. Thus, most of these crustaceans were absent from open-slope sites. Higher coral diversity may equate to a higher number of niches available to crustaceans, thereby enabling more species to coexist.

Broad-scale habitat and depth also influenced abundances of *C*. *quinquedens* across the region. Abundance estimates ranged from 0.005 to 2.087 individuals 10 m^-2^. Abundances were highest in shallow, open slope/landslide scar and inter-canyon areas and lowest in deeper canyon environments. However, many individuals were likely missed at each site as we observed numerous individuals digging into sediments and entering burrows. Thus, abundances are likely underestimated. In general, abundances declined with depth, with few individuals observed at depths > 1400 m (Fig 8E). However, depth was not the only influencing factor on abundance of red crabs, as few individuals were observed at shallower depths (< 1400 m) in canyon environments. Perhaps the steep, vertical walls, and rugged seafloor in canyon environments inhibit this species from digging and/or foraging in soft sediments [116]. Additionally, a peak in abundance (1.269 individuals 10 m^-2^) was estimated at a cold seep site (NE Seep 2) at depths of 1053 to 1139 m. In all cold seep environments, the majority of individuals were observed on the periphery of mussel beds. However, several individuals were also observed in crevices in authigenic carbonates (Veatch Seeps) and lying directly on living mussels. *Chaceon* sp. has previously been documented as vagrant members of cold seeps [117], which may locally increase food supply.

#### Additional Factors Likely Impacting Assemblage Structure

Although the environmental variables examined in the present study explain a large portion of the variation (67–71%) in assemblage structure for each taxonomic group (DistLM, BEST), additional environmental factors not quantified in this study likely influence the assemblage patterns observed. Current flow, internal waves, downwelling and tidal forcing affect the distribution and abundance of cold-water corals and other sessile suspension feeders [118–121]. In particular, accelerated currents over rugged, topographic highs promote feeding efficiency and/or larval supply [118, 121]. These oceanographic mechanisms in combination with other factors such as aragonite saturation depth [122, 123] and dissolved organic material [122] may further influence the patterns in coral assemblage structure observed in this study. Additionally, it is possible that mortality from predation events (e.g, pycnogonids [89], seastars, [43]) could influence local-scale alpha diversity of corals.

We hypothesize that the underlying geological processes that promote or inhibit colonization (i.e., habitat availability and suitability) across canyons, slopes, and seamounts are also important in influencing coral assemblage structure. Canyon wall failures, sedimentation and landslides (i.e., unstable habitat) may lead to increased mortality and reduced recruitment success in certain areas. In contrast, more stable features likely facilitate the colonization and maintenance of sessile species. For example, species richness (28 species) was highest in Nygren Canyon (2 dives, 678–1590 m, S1 Table, Table 2). The canyon walls in Nygren appeared to be highly stable, as suggested by the presence of Fe-Mn oxide coating and heavy colonization of attached fauna. In contrast, fewer species (16 species; 2 dives, 846–1110 m) were observed in Alvin Canyon. Here, the canyon walls were highly eroded, often mantled by debris aprons and numerous sediment/water flow chutes along the canyon walls (Fig 2). Therefore, geological disturbances may increase coral mortality and reduce recruitment success. Relative stability of the underlying geology, in combination with small-scale oceanographic mechanisms, may thus drive patterns of coral alpha diversity and distribution observed along canyon walls.

For fishes and crustaceans, food availability and microhabitat diversity could explain additional variation. Input of organic matter could have a profound effect on assemblage structure by changing the population densities of certain organisms both temporally and spatially [124]. These population changes could thus affect the amount and intensity of biotic interactions (e.g., predation, competition, symbioses), which could then further alter assemblage patterns. Microhabitat utilization is also an important component in faunal assemblage structure on the continental slope [125]. Some species of fishes and invertebrates are tightly associated with corals and/or structurally complex terrain [40, 94, 107, 126]. Additionally, sediment composition (e.g., clay, mud, shell hash) may influence burrowing and feeding behavior of crustaceans [125]. Microhabitats can also be characterized by abundance and type of coral species present. The presence of certain coral species may considerably influence crustacean assemblage patterns. For example, the squat lobster *Uroptychus* sp. (Fig 4G) was only observed on the black coral *Parantipathes* sp. In contrast, *Munidopsis* spp. utilized a variety of coral species, particularly those with structurally complex morphologies, as habitat. Species-specific relationships of corals and invertebrates require further investigation [37, 42]. In conclusion, various degrees of habitat heterogeneity, including rugosity, high or low relief profile, abundance and type of corals, hard or soft substrates, stability and origin of canyon substrate, and sediment composition likely influence local assemblage structure within the NEUS region.

### Conservation Considerations

**Fig 9:**
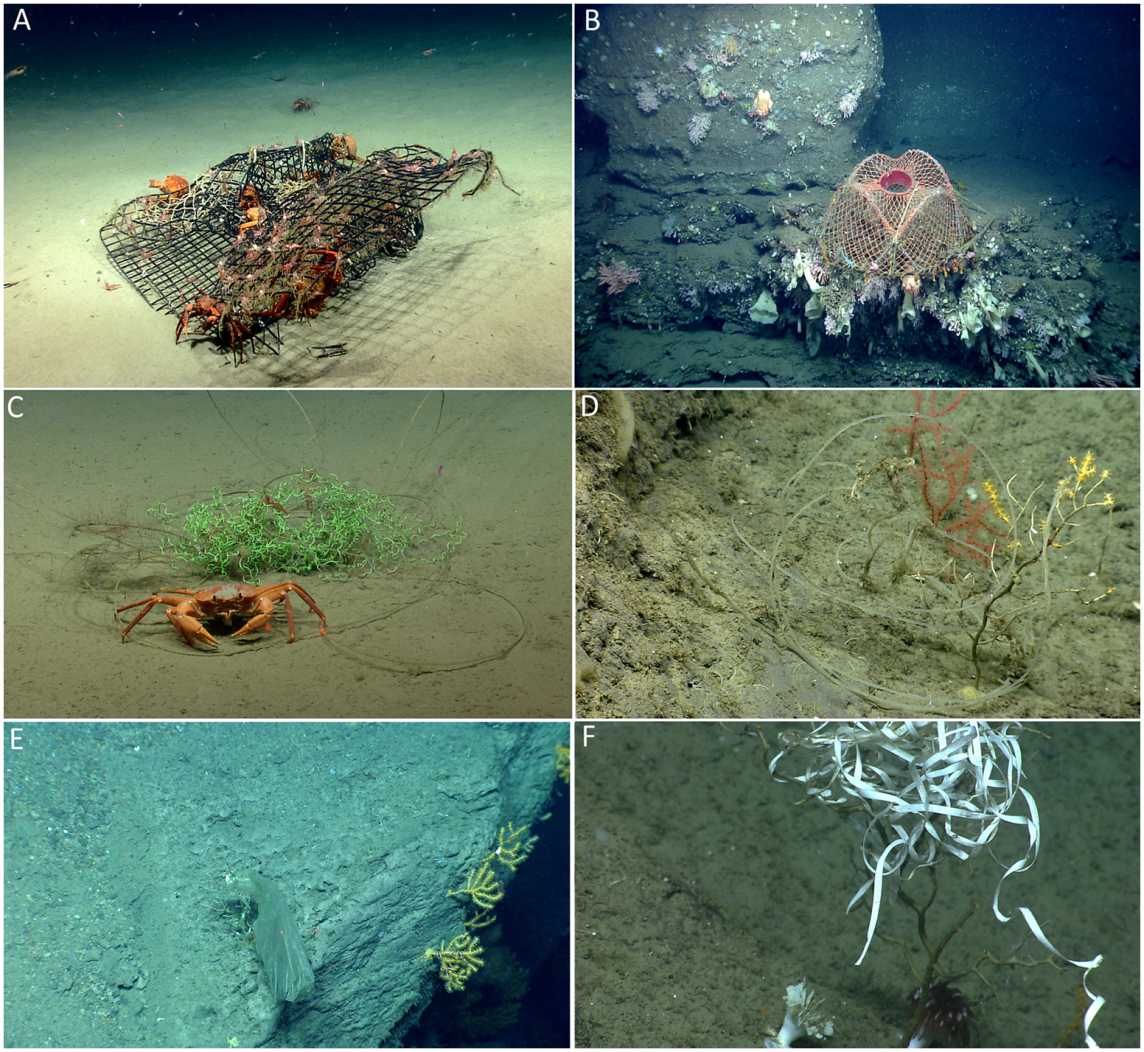
Examples of marine litter. (A) A ghost fishing trap with red crabs (*Chaceon quinquedens*) and banded shrimp (USGS4, 575 m). (B) A ghost fishing trap in Nygren-Heezen inter-canyon area (697 m). (C) Fishing line with *C*. *quinquedens* at USGS1 (782 m). (D) Fishing line wrapped around a partially dead *Paramuricea* sp. in Oceanographer Canyon (1222 m). (E) Remnants of a balloon wrapped around a dead coral skeleton in Hydrographer Canyon (1376 m). (F) Ribbon wrapped around a dead coral skeleton in Oceanographer Canyon (1220 m).

**Fig 10:**
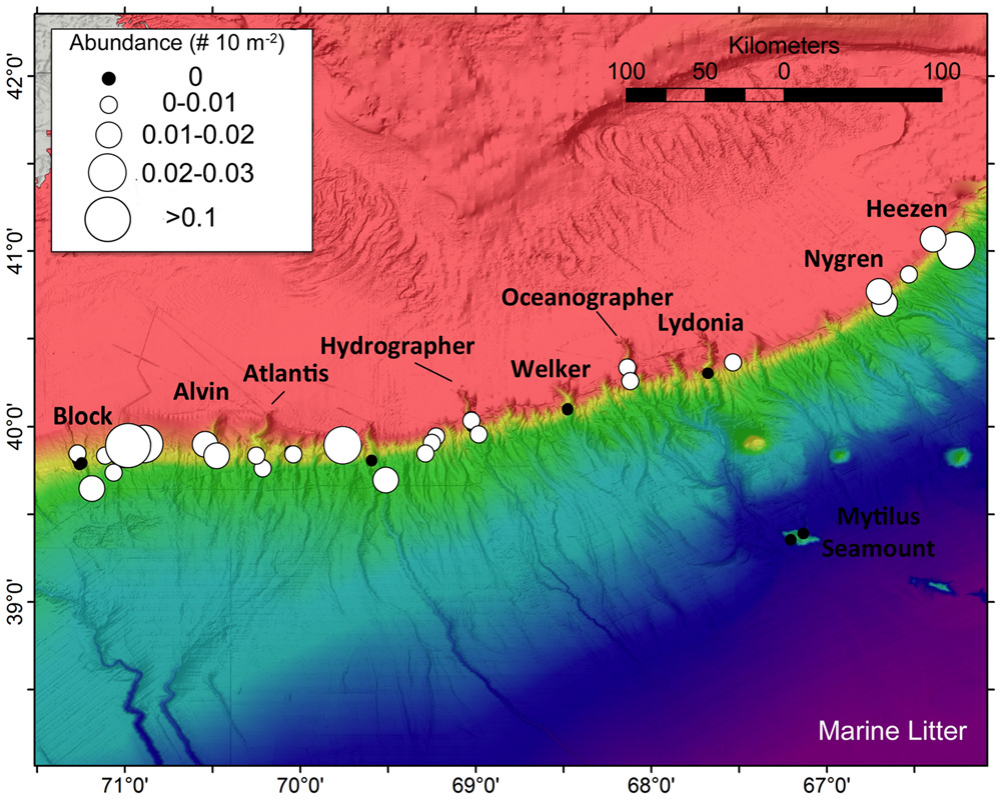
Abundance estimates of marine litter observed during each dive.

Signs of human disturbance along the NEUS continental margin were evident. Marine litter (140 items) was encountered on 81% of the dives throughout the depth range and region surveyed (S1 Table, Figs 9 and 10). Litter included derelict fishing gear (traps, monofilament line, hooks and reels) and other debris (e.g., soda cans, glass bottles, balloons, rugs, plastic bags) (Fig 9). Estimated quantities of marine litter ranged from 0.002 to 0.130 items 10 m^-2^, with the highest estimates found in an un-named slope canyon (S1 Table, Fig 10). At least 12 coral colonies were entangled with debris. One ghost-fishing trap was observed lying on top of at least three octocoral species and one dead scleractinian (Fig 9B). A dead scleractinian colony and two piles of dead scleractinian, skeleton rubble were entangled with monofilament line. Additionally, five octocoral colonies (*Paramuricea* spp., and *Thouarella*? *grasshoffi*) were entangled with either monofilament line or balloon remnants, resulting in varying degrees of exposed, dead skeleton (Fig 9D–9F). These observations demonstrate that litter can impact corals in the deep sea. Many of these corals can be long lived. For example, *Paramuricea biscaya* from the Gulf of Mexico has been documented to live for more than 600 years [127].

Cold-water coral ecosystems along the NEUS continental slope may be particularly vulnerable to disturbances. With additional fishing activity in inter-canyon areas and on the upper slope [51], the high potential for hydrocarbon exploration and extraction [4], and the proximity to a populated area along the U.S. coast, anthropogenic disturbances to benthic communities along the NEUS continental margin will likely increase. Yet, these ecosystems are biodiversity hotspots [87]. Cold-water corals are long-lived and slow-growing [127–129] and likely require decades to centuries to recover from anthropogenic disturbances [130]. Thus, areas within this region off the NEUS may respond positively to effective protection measures.

## Conclusions and Further Research

This study provided much needed data and baseline observations of the geology and biodiversity in underexplored areas on the NEUS continental margin. Our surveys indicated that submarine canyons, inter-canyon sites and the flanks of seamounts along the NEUS are geologically dynamic areas that respond to a wide variety of physical, chemical, and biological processes. Furthermore, observations of the exposed lithologies along the walls of the submarine canyons provide a glimpse into the long geologic history of the NEUS continental margin that has only been seen via a limited number of submersible dives, geophysical imaging and widely spaced drill holes and grab samples. The visual surveys from this expedition provide a basis for the development of future investigations on both short and long-term processes influencing landscape ecology, continental margin geology and geohazard analysis in the deep sea.

Initial assessments of biodiversity revealed the importance of both depth and broad-scale habitat features in shaping the patterns of species richness and composition observed throughout this region. Although factors (e.g., pressure, temperature, salinity) that co-vary with depth are important in shaping community structure throughout the deep sea, how and where these factors impact community structure depend upon the region and the taxonomic group examined. We found that species richness (alpha diversity) declined with depth for decapod crustaceans and demersal fishes, but not for corals. These contrasting patterns in species richness are likely a result of differing evolutionary processes, such as the rapid diversification of corals in deep waters [88], and ecological requirements shaping faunal diversity in the deep sea. However, we did find that species turnover (beta diversity) occurred on the middle to lower slope for all taxonomic groups. Turnover in assemblage structure on the middle-lower slope closely matches depths where boundaries between water masses occur. For instance, Labrador Sea water characterized by temperatures of 3–4°C is generally at 1300 to 2500 m; the shallower portion of the deep western boundary current water characterized by temperatures of 4–5°C is at 700 to 1300 m [131]. Finally, our results indicated that species composition and/or diversity differed among soft-sedimented open-slope, cold-seep, and canyon sites. Although assemblage structure needs to be further refined to reflect specific microhabitats observed in this region, the broad-scale habitats as defined herein do not appear to be functionally equivalent for any taxonomic group. Abundance estimates for species examined in this study also support this conclusion.

This exploration along the NEUS continental margin demonstrates the need for further investigations to increase our understanding of community structure within and around diverse canyon ecosystems. Further information regarding microhabitats, current flow, and organic matter input within the broad-scale habitat features is necessary to improve our understanding of the observed patterns in community structure. Examination of other taxa (e.g., sponges, echinoderms and octopods) and coral-invertebrate relationships within and around canyons warrant additional investigation. Also, voucher specimens are required to confirm species identifications presented in this study. Future survey efforts need to incorporate a rigorous sampling design by including additional locations and depths across habitat features to refine the understanding of faunal differences. Additional environmental data (e.g., current regime, organic input) are required to better determine the mechanistic factors that affect the diversity, abundance, and distribution of fauna associated with these deep-sea habitats.

## Supporting Information

S1 Fig Species richness by ROV distance. Species richness by distance travelled by the ROV for (A) corals. (B) demersal fishes. (C) crustaceans. Best-fit linear trend lines are included.
(PDF)

S2 Fig Examples of reproductive activity in canyon environments. (A) *Graneledone verrucosa* guarding eggs. (B) Bobtail squid eggs in sponge cavities. (C) Catshark (Scyliorhinidae) egg cases attached to octocorals. (D) Skate (Rajiidae) egg case on the seafloor.
(PDF)

S1 Table Site data. Location and environmental data for dives conducted during the 2013 northeast US canyon expedition.
(XLSX)

S2 Table Corals. Coral presence documented during each dive conducted during the 2013 northeast US Canyons Expedition.
(XLSX)

S3 Table Demersal fishes. Demersal fish presence documented during each dive conducted during the 2013 northeast US Canyons Expedition.
(XLSX)

S4 Table Decapod crustaceans. Decapod crustacean presence documented during each dive conducted during the 2013 northeast US Canyons Expedition.
(XLSX)

S5 Table Abundance estimates. Abundances estimated per dive for five dominant species.
(XLSX)
