# Supplementary figures and images for: Exploration of the Canyon-Incised Continental Margin of the Northeastern United States Reveals Dynamic Habitats and Diverse Communities
Source: PLoS One. 2015 Oct 28;10(10):e0139904. doi: 10.1371/journal.pone.0139904 (PMC4624883; doi:10.1371/journal.pone.0139904)

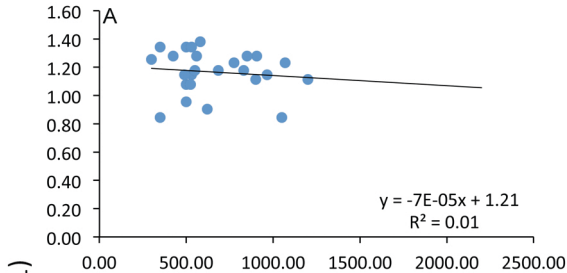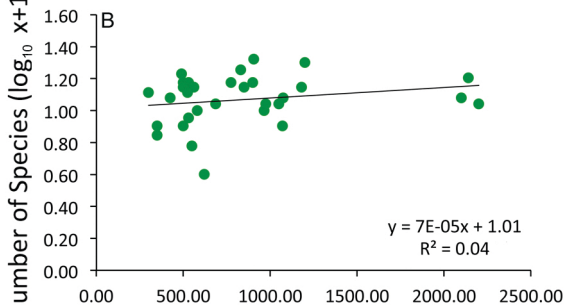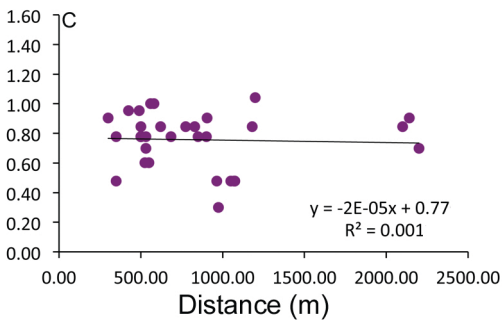

Supplement: S1 Fig — Species richness by distance travelled by the ROV for (A) corals. (B) demersal fishes. (C) crustaceans. Best-fit linear trend lines are included. (PDF) [file pone.0139904.s001.pdf]

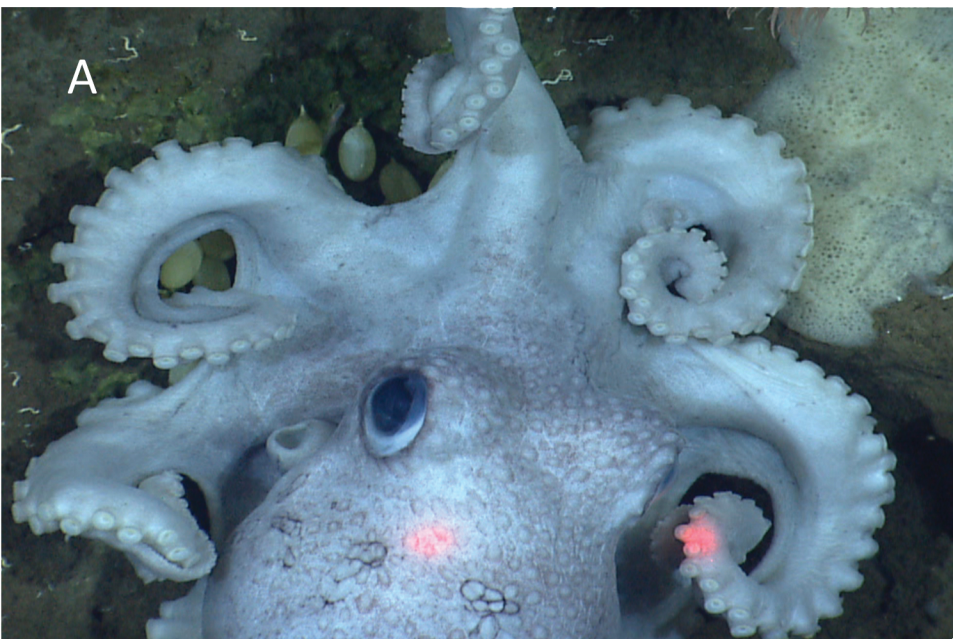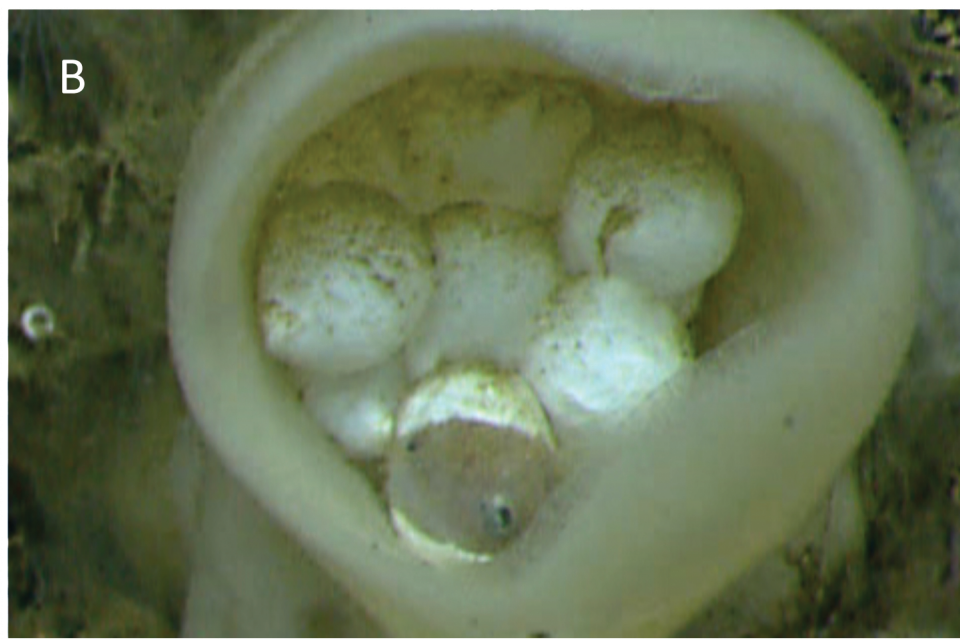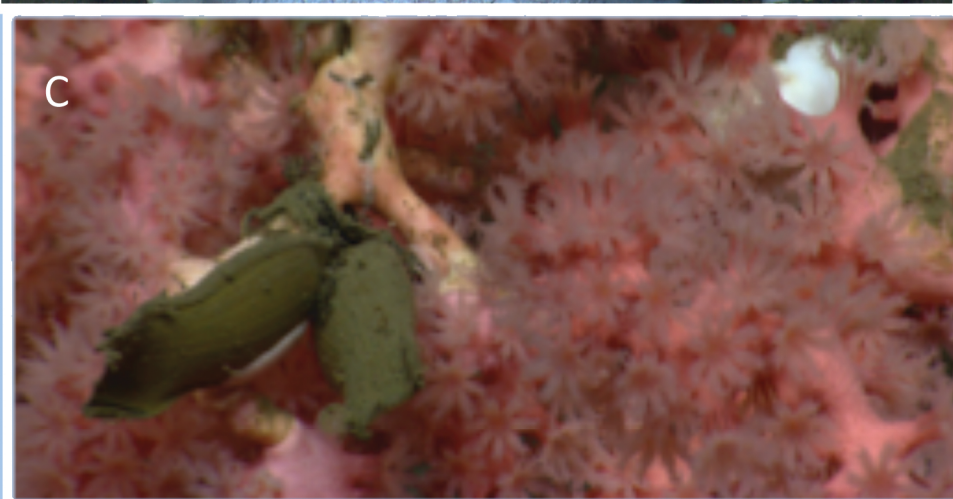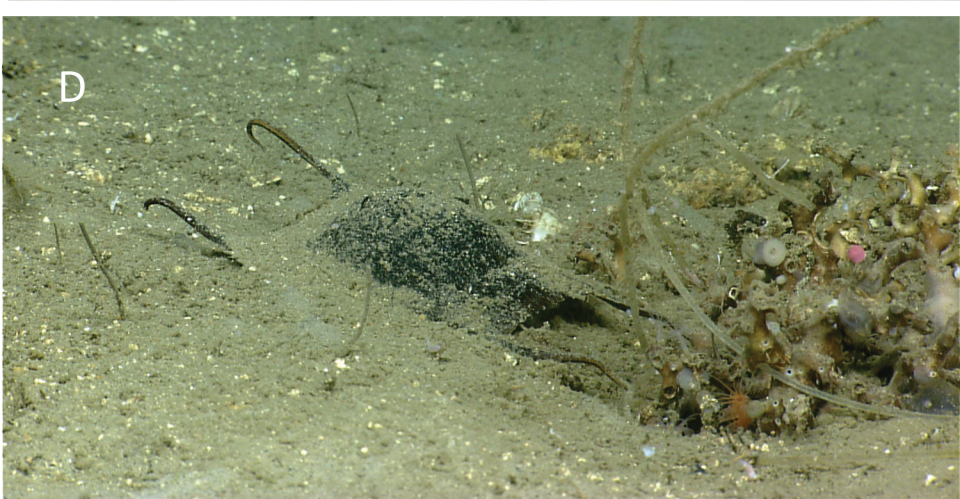

Supplement: S2 Fig — (A) Graneledone verrucosa guarding eggs. (B) Bobtail squid eggs in sponge cavities. (C) Catshark (Scyliorhinidae) egg cases attached to octocorals. (D) Skate (Rajiidae) egg case on the seafloor. (PDF) [file pone.0139904.s002.pdf]
